# Tenecteplase in the Extended 4.5–24-Hour Window for Acute Ischemic Stroke: An Updated Meta-Analysis of RCTs with EVT-Stratified Subgroup Analysis

**DOI:** 10.3390/healthcare14111470

**Published:** 2026-05-26

**Authors:** Sadia Qazi, Arsalan Ahmed, Mazhar Ali, Muhammad Usman Iqbal, Eshal Atif, Zain Ali, Abdullah Imtiaz, Nabahat Shafi, Muhammad Hassan Imtiaz, Mohammad Dawar Zahid, Muhammad Sharjeel Abbas, Muhammad Atif Mazhar

**Affiliations:** 1 Department of Anatomy, College of Medicine, Alfaisal University, Riyadh 11533, Saudi Arabia; 2 Medical College, Liaquat University of Medical Health Sciences, Jamshoro 76090, Pakistan; arsalanahmed7722@gmail.com; 3 Department of Medicine, Khyber Medical College, Khyber Medical University, Peshawar 25120, Pakistan; mazharaliuss@gmail.com; 4 Department of Medicine, King Edward Medical University, Lahore 54000, Pakistan; usmaniqbal044@gmail.com; 5 College of Medicine, Alfaisal University, Riyadh 11533, Saudi Arabia; eatif@alfaisal.edu; 6 Department of Medicine, Wah Medical College, National University of Medical Sciences (NUMS), Rawalpindi 47010, Pakistan; zainalimd23@gmail.com (Z.A.); abdullahimtiaz5240@gmail.com (A.I.); 7 Dow Medical College, Dow University of Health Sciences, Karachi 74200, Pakistan; nabahatshafi1@gmail.com; 8 Shaikh Khalifa Bin Zayed Al Nahyan Medical and Dental College, Muslim Town, Lahore 53720, Pakistan; hk6016500@gmail.com; 9 Medical College, Aga Khan University, Stadium Road Campus, Karachi 74800, Pakistan; mohammaddawar.zahid@scholar.aku.edu; 10 Department of Medicine, Gomal Medical College, Khyber Medical University, Peshawar 29111, Pakistan; sharmehw1214@gmail.com

**Keywords:** tenecteplase, acute ischemic stroke, extended window, endovascular thrombectomy, thrombolysis, meta-analysis

## Abstract

**Background:** The efficacy of tenecteplase for acute ischemic stroke (AIS) beyond 4.5 h remains uncertain, particularly across care pathways with and without endovascular thrombectomy (EVT). We performed an updated systematic review and meta-analysis using an EVT-stratified framework. **Methods:** PubMed, Embase, Scopus, and the Cochrane Library were searched through February 2026 for randomized controlled trials comparing tenecteplase with control in imaging-selected patients with AIS presenting 4.5–24 h from last known well. The primary outcome was excellent functional outcome (mRS 0–1) at 90 days. Secondary outcomes were good functional outcome (mRS 0–2), recanalization, early neurological improvement, symptomatic intracranial hemorrhage, and 90-day mortality. Random-effects models with Hartung–Knapp adjustment were used. Subgroup analyses by EVT availability were interpreted as exploratory because of the limited number of trials. **Results:** Five trials including 1844 patients were analyzed. Tenecteplase improved excellent functional outcome (RR 1.25, 95% CI 1.10–1.42; *p* = 0.0005) with no heterogeneity (I^2^ = 0%) and no interaction by EVT status (*p*-interaction = 0.961). Good functional outcome was not significantly different overall (RR 1.10, 95% CI 0.97–1.24; *p* = 0.135). Significant subgroup interactions were observed for recanalization (*p*-interaction = 0.004) and early neurological improvement (*p*-interaction = 0.002), with benefits concentrated in non-EVT settings. However, the larger effect on recanalization did not translate proportionally into functional recovery, supporting separation of vessel-opening outcomes from patient-centered outcomes. Symptomatic intracranial hemorrhage showed a nonsignificant increase in four estimable studies (RR 1.88, 95% CI 0.94–3.78; *p* = 0.074), whereas 90-day mortality did not differ significantly (RR 1.11, 95% CI 0.85–1.43; *p* = 0.43). **Conclusions:** In imaging-selected AIS presenting 4.5–24 h after onset, tenecteplase improved excellent functional outcome irrespective of EVT availability, while benefits for recanalization and early neurological improvement were largely confined to non-EVT settings. Because recanalization is an intermediate endpoint, these findings should not be interpreted as proof of a proportional clinical benefit. Future extended-window trials should specify EVT status.

## 1. Introduction

Acute ischemic stroke (AIS) remains a major global cause of death and disability, with a substantial healthcare burden worldwide [1]. Intravenous thrombolysis is a central reperfusion strategy for AIS; however, its benefits are highly time-dependent. The modern 4.5-h treatment benchmark was established by ECASS III, which demonstrated the benefit of alteplase in carefully selected patients treated beyond 3 h and up to 4.5 h after symptom onset [2]. However, many patients present beyond this conventional window, making imaging-based selection increasingly important for identifying salvageable brain tissue rather than relying solely on the time from onset.

Tenecteplase (TNK), a genetically modified variant of recombinant tissue plasminogen activator, offers several pharmacologic and practical advantages over alteplase, including greater fibrin specificity, a longer half-life, and greater resistance to plasminogen activator inhibitor-1, allowing for single-bolus administration [3]. These properties may support more efficient clot lysis and simplify acute stroke workflows, particularly in emergency departments, transfer pathways, and “drip-and-ship” systems, where infusion-based alteplase can be logistically more difficult. Within the standard treatment window, randomized trials have supported TNK as an effective alternative to alteplase [4,5]. A prior meta-analysis pooled these different settings, which may have obscured the clinically important effect modification by EVT availability [6]. Nevertheless, pharmacologic advantages do not automatically imply superior clinical outcomes because functional recovery depends not only on vessel recanalization but also on infarct core size, collateral circulation, time to reperfusion, microvascular perfusion, hemorrhagic transformation, and post-stroke care.

This question has become increasingly important in the thrombectomy era. Endovascular thrombectomy (EVT) improves outcomes in selected patients with large vessel occlusions presenting up to 24 h after onset [7]. However, access to EVT remains uneven across regions and health systems, creating a persistent gap between trial-supported efficacy and real-world availability [8]. Consequently, many patients, particularly those presenting to non-thrombectomy centers or resource-constrained systems, may still depend primarily on pharmacologic reperfusion [8]. In EVT-eligible patients, TNK may function mainly as bridging thrombolysis before thrombectomy, whereas in non-EVT settings, it may represent the principal reperfusion strategy. Therefore, EVT availability is not a minor procedural detail; it may modify the apparent efficacy of TNK and should be examined separately in future studies.

Recent randomized controlled trials evaluating TNK in the 4.5–24-h window have produced mixed results across distinct care pathways. The ROSE-TNK trial suggested the feasibility of this approach in MRI-selected patients [9]. TIMELESS, conducted largely in an EVT-permitted population, showed neutral results [10]. In contrast, TRACE-III demonstrated benefits in patients treated without thrombectomy [11]. CHABLIS-T II [12] and the more recent OPTION trial [13] further expanded the evidence base, but they also increased clinical heterogeneity with respect to vessel status, imaging selection, and treatment pathways. Prior syntheses have generally pooled these differing settings, which may obscure clinically important effect modifications by EVT availability [6]. This distinction is clinically important because a stronger effect on recanalization or reperfusion may not translate proportionally into 90-day functional independence, especially when irreversible tissue injury, distal embolization, incomplete microvascular reperfusion, or delayed definitive treatment limits neurological recovery.

Simultaneously, the evidence base for extended-window thrombolysis with alteplase has also advanced. The WAKE-UP [14] and EXTEND [15] trials showed that imaging-guided thrombolysis can improve outcomes in selected late-presenting patients, and an individual patient data meta-analysis further reinforced the principle that tissue status, rather than clock time alone, can guide reperfusion decisions in this setting [16]. Advanced imaging approaches, including DWI-FLAIR mismatch, perfusion-core mismatch, vascular imaging, and collateral assessment, may help identify patients most likely to benefit from late thrombolysis and may also explain why angiographic improvement and clinical recovery can diverge.

Against this background, we conducted an updated systematic review and meta-analysis of randomized trials evaluating TNK in AIS treated 4.5–24 h after onset, with a prespecified EVT-stratified analysis. We aimed to determine whether EVT availability modifies the treatment effect of TNK, whether TNK improves 90-day functional outcomes, recanalization, and early neurological improvement, and whether any efficacy signal is offset by increased risks of symptomatic intracranial hemorrhage, systemic bleeding, or death. Because available trials differ in treatment pathway, imaging-selection criteria, and regional stroke-system context, we interpreted EVT-stratified findings cautiously and treated subgroup results as clinically informative but not definitive evidence of differential efficacy.

## 2. Methods

### 2.1. Protocol and Reporting Standards

This systematic review and meta-analysis was conducted in accordance with the Preferred Reporting Items for Systematic Reviews and Meta-Analyses (PRISMA) 2020 statement [17], and the checklist is presented in Supplementary Table S1. The study protocol was prospectively registered with the PROSPERO database (CRD420261347008). The primary objective was to evaluate the efficacy and safety of tenecteplase (TNK) in the extended 4.5–24-h window for acute ischemic stroke (AIS), with a specific focus on effect modification by endovascular thrombectomy (EVT) availability. Because EVT eligibility, imaging-selection strategy, and baseline stroke severity may influence both reperfusion and functional recovery, the review was designed to separately examine EVT-permitted and non-EVT settings where trial-level data were available.

### 2.2. Search Strategy and Eligibility Criteria

A systematic search was performed in PubMed, the Cochrane Library, Embase, and Scopus from database inception to February 2026. We used a broad combination of MeSH terms and free-text keywords for tenecteplase and acute ischemic stroke; the extended window criterion was applied during eligibility screening rather than as a search filter. The full search strings and database-specific results are presented in Supplementary Table S2.

Studies were eligible if they were randomized controlled trials (RCTs) enrolling adult patients with AIS presenting 4.5–24 h after the last known well, comparing TNK with control treatment, including placebo, standard care, or standard medical treatment, and using advanced imaging for treatment selection, such as DWI-FLAIR mismatch or perfusion-based paradigms. We excluded non-randomized studies, observational cohorts, and abstracts lacking sufficient quantitative data for effect size calculation.

### 2.3. Study Selection and Data Extraction

**Figure 1:**
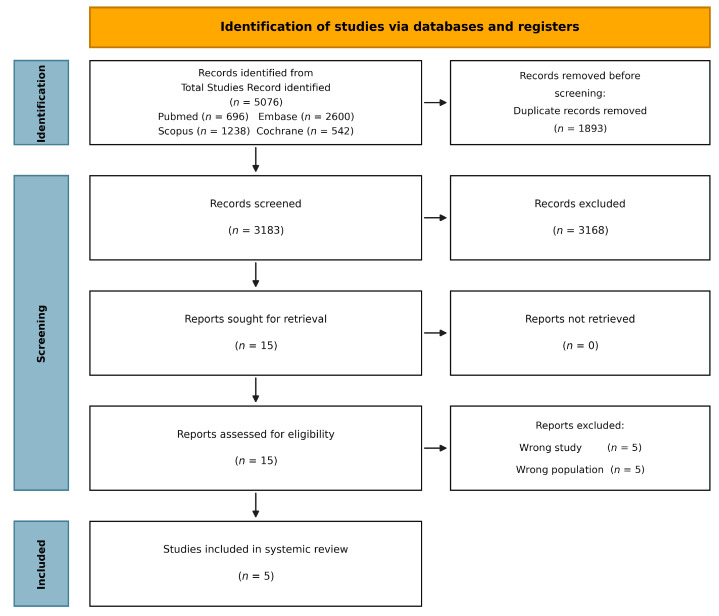
PRISMA 2020 flow diagram of the systematic review and study selection process. Data were extracted using a standardized template, including trial identifiers, patient demographics, baseline NIHSS scores, imaging modalities, EVT utilization rates, and numerical data for all prespecified efficacy and safety outcomes. Additional extracted variables included sample size, country or region of recruitment, TNK dose, comparator strategy, time-window criteria, occlusion site information when available, imaging-selection paradigm, EVT permissibility, actual EVT utilization where reported, and baseline clinical characteristics relevant to treatment response. Where available, outcome data were extracted separately for EVT-permitted and non-EVT settings.

Two reviewers independently screened the titles and abstracts, followed by a full-text assessment against the inclusion criteria. Discrepancies were resolved by a third reviewer. The selection process is summarized in the PRISMA flowchart (Figure 1).

### 2.4. Risk of Bias and Certainty of Evidence

Methodological quality was assessed using the Cochrane Risk of Bias 2 tool (RoB 2) [18] across five domains: randomization process, deviations from intended interventions, missing outcome data, measurement of the outcome, and selection of reported results. The certainty of the evidence for each outcome was assessed using the Grading of Recommendations Assessment, Development, and Evaluation (GRADE) framework [19]. Certainty ratings considered the risk of bias, inconsistency, indirectness, imprecision, and publication bias. Particular attention was given to inconsistency and imprecision, where estimates were based on few trials or events or where treatment effects varied according to the EVT pathway.

### 2.5. Statistical Analysis and Heterogeneity

Quantitative synthesis was performed using R software version 4.4.2 [20] and the meta package [21]. Random-effects models were used for all analyses to account for the anticipated clinical heterogeneity. Between-study variance (tau-squared) was estimated using the restricted maximum-likelihood method, with confidence intervals for tau-squared derived using the Q-profile method. To provide conservative estimates, given the small number of trials, the Hartung–Knapp adjustment was applied to all pooled analyses [22].

Effect sizes were expressed as risk ratios (RRs) with 95% confidence intervals (CIs). Statistical heterogeneity was evaluated using Cochran’s Q test and quantified with the I-squared statistic [23], where 25%, 50%, and 75% were interpreted as low, moderate, and substantial heterogeneity, respectively. Efficacy outcomes included excellent functional outcome, defined as mRS 0–1 at 90 days; good functional outcome, defined as mRS 0–2 at 90 days; recanalization or reperfusion, as defined by the individual trials; and early neurological improvement, generally assessed by NIHSS change within 24–72 h. Safety outcomes included symptomatic intracranial hemorrhage, 90-day mortality, and moderate or severe systemic bleeding. Recanalization and reperfusion outcomes were considered intermediate or surrogate endpoints and were analyzed separately from the final functional outcomes.

### 2.6. Subgroup and Sensitivity Analyses

The primary hypothesized source of heterogeneity was EVT availability. We performed prespecified subgroup analyses comparing non-EVT settings, defined as no planned or available thrombectomy, with EVT-permitted settings, defined as thrombectomy or bridging therapy allowed by the trial protocol. Subgroup classification was based on protocol-level EVT permissibility, rather than actual EVT utilization. Accordingly, OPTION was categorized as EVT-permitted because rescue EVT was allowed by the protocol, although observed EVT use was low (1.3%). Effect modification was evaluated using the chi-square test for subgroup interactions. Because subgroup interaction tests are commonly underpowered when the number of included studies is small, non-significant interaction results were not interpreted as evidence of equivalent treatment effects across EVT and non-EVT settings. These analyses were exploratory and hypothesis-generating.

To assess robustness, we conducted a suite of diagnostic analyses [24]: leave-one-out influence analysis, Graphical Display of Study Heterogeneity (GOSH), Baujat plots, drapery plots, and funnel plot assessment of small-study effects. These diagnostic assets are organized in the supplement as Supplementary Figures S1–S10 (Block A: Functional Outcomes), Supplementary Figures S11–S20 (Block B: Revascularization and Recovery), and Supplementary Figures S21–S35 (Block C: Safety Outcomes). These diagnostic analyses were used to assess whether the pooled estimates were disproportionately influenced by individual trials, small-study effects, or between-study heterogeneity. Differences in EVT permissibility, actual EVT utilization, and imaging-selection strategies were considered when interpreting heterogeneity across outcomes.

## 3. Results

### 3.1. Study Selection and Quality Appraisal

The systematic literature search yielded 5080 records from PubMed, Embase, Scopus, and Cochrane Library. After removing duplicates and screening 2776 records, 15 full-text articles were evaluated for their eligibility. Five randomized controlled trials (RCTs) met the final inclusion criteria: ROSE-TNK [9], TIMELESS [10], TRACE-III [11], CHABLIS-T II [12], and OPTION [13]. The study selection process is summarized in the PRISMA flow diagram (Figure 1). Adherence to the reporting standards is documented in the PRISMA 2020 checklist (Supplementary Table S1), and the comprehensive search strategy for each database is available in Supplementary Table S2. No nonrandomized studies were included in the quantitative synthesis.

**Figure 2:**
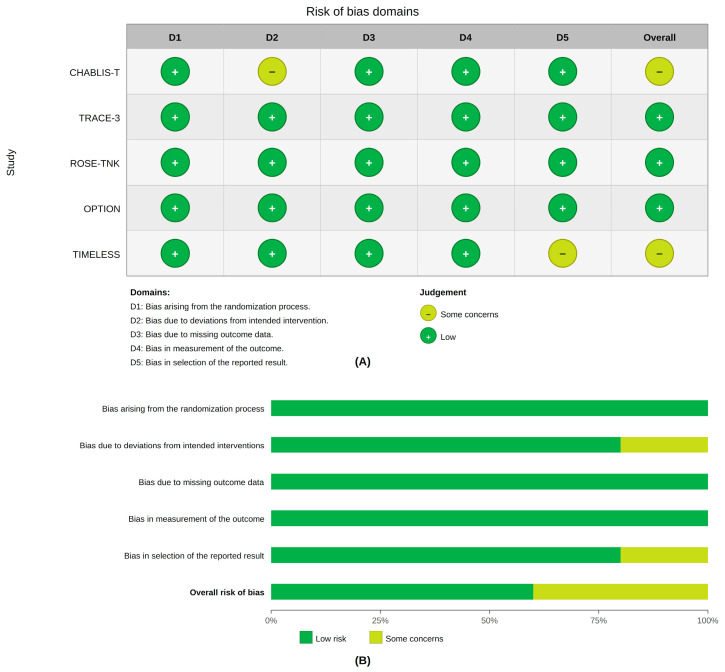
Risk of bias assessment using the RoB 2. (**A**) Domain-level judgments for each trial included. (**B**) Proportion of studies rated as low risk or having some concerns across the five RoB 2 domains.

Risk of bias assessment showed that three trials were judged to be at low overall risk of bias, whereas two trials had some concerns (Figure 2). These concerns were mainly related to deviations from the intended interventions in CHABLIS-T II and the selection of the reported results in TIMELESS. No included trial was judged to have a high overall risk of bias.

### 3.2. Study and Participant Characteristics

The five trials enrolled a total of 1844 randomized patients across China, the United States, and Canada between 2023 and 2026: ROSE-TNK [9], TIMELESS [10], TRACE-III [11], CHABLIS-T II [12], and OPTION [13]. Four of the five included trials were conducted in China [9,11,12,13], whereas TIMELESS was conducted in the United States and Canada [10]. All trials utilized advanced imaging, such as MRI-based DWI-FLAIR mismatch or CT/MRI perfusion, to identify patients with salvageable tissue in the 4.5–24-h window [9,10,11,12,13]. Participants generally presented with NIHSS-defined disabling stroke, with most trials using NIHSS ranges of approximately 6–25, whereas TIMELESS enrolled patients with large vessel occlusion selected by perfusion imaging [9,10,11,12,13]. Trial-level endovascular thrombectomy (EVT) utilization varied significantly, ranging from 0% in ROSE-TNK and TRACE-III [9,11] to 77.4% in TIMELESS [10]. A total of 1841 patients were included in the primary efficacy analysis. The full baseline characteristics of the included trials are presented in Table 1.

The baseline profiles differed clinically across the trials. TIMELESS enrolled an older cohort with large vessel occlusion and high EVT utilization [10], whereas ROSE-TNK and TRACE-III were non-EVT studies [9,11]. OPTION enrolled patients with non-large-vessel occlusion and very low rescue EVT use [13]. CHABLIS-T II enrolled patients with large- or medium-vessel occlusions selected by perfusion imaging and included substantial EVT use [12]. These differences indicate that the pooled population included distinct treatment pathways rather than a single homogeneous extended window AIS population.

Across treatment arms within individual trials, baseline NIHSS scores were generally similar, although differences in age, sex distribution, occlusion status, imaging paradigm, and EVT availability varied across the studies [9,10,11,12,13]. These trial-level differences were considered when interpreting the heterogeneity and subgroup findings.

### 3.3. Functional Outcomes

A comprehensive summary of all efficacy and safety outcomes, including pooled risk ratios and subgroup interaction tests, is presented in Table 2. A summary of the pooled results for all outcomes and the GRADE certainty assessment are presented in Supplementary Table S3 and Table S4, respectively. Functional outcomes were interpreted separately from recanalization and early neurological improvement because vessel opening and early clinical changes do not necessarily translate into sustained 90-day functional independence.

**Figure 3:**
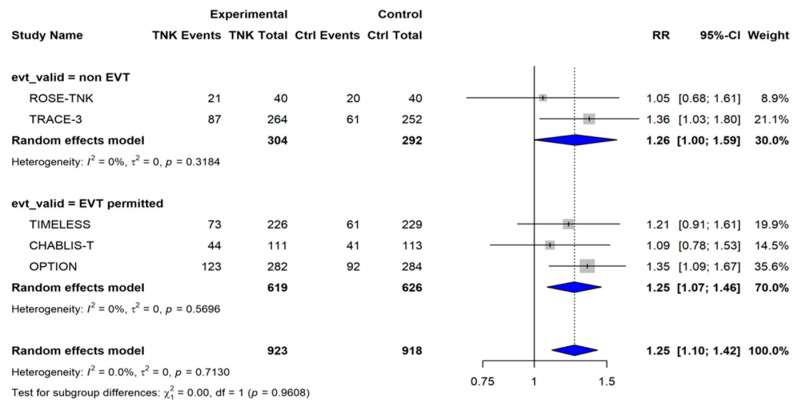
Forest plot of excellent functional outcome stratified by EVT status. Gray squares represent study-specific risk ratios (RRs), with square size proportional to study weight; horizontal black lines indicate 95% confidence intervals (CIs). Blue diamonds represent pooled random-effects estimates for each subgroup and the overall analysis, with diamond width indicating the 95% CI. The solid vertical black line indicates the line of no effect (RR = 1), and the dashed vertical black line indicates the overall pooled effect estimate. EVT, endovascular thrombectomy; TNK, tenecteplase; RR, risk ratio; CI, confidence interval.

Excellent functional outcome (mRS 0–1): A pooled analysis of five studies (*n* = 1841) showed that tenecteplase significantly improved the rate of excellent functional outcome at 90 days (RR 1.25, 95% CI 1.10–1.42; *p* = 0.0005), with no observed heterogeneity (I-squared = 0%, *p* = 0.713) (Figure 3). This benefit was consistent across both the non-EVT (RR 1.26) and EVT-permitted (RR 1.25) subgroups, with no significant interactions (*p* = 0.961). Because the point estimates were nearly identical across EVT-defined subgroups and heterogeneity was absent, this outcome showed the most stable functional signal in our analysis. Robustness diagnostics, including sensitivity and influence plots for this outcome, are provided in Supplementary Figures S1–S5 (Block A).

**Figure 4:**
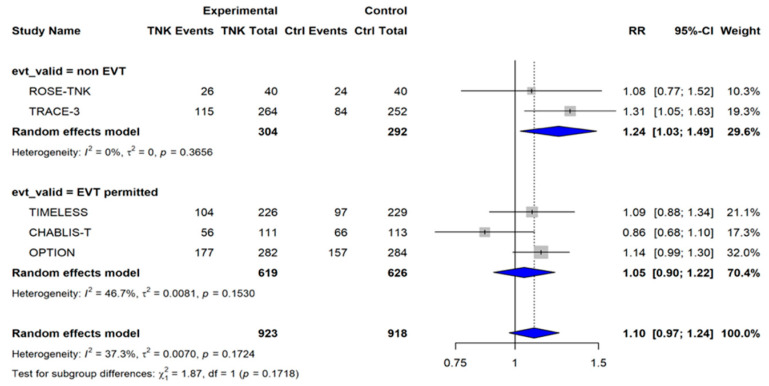
Forest plot of good functional outcome stratified by EVT status (mRS 0–2) at 90 days. Gray squares represent study-specific risk ratios (RRs), with square size proportional to study weight; horizontal black lines indicate 95% confidence intervals (CIs). Blue diamonds represent pooled random-effects estimates for each subgroup and the overall analysis, with diamond width indicating the 95% CI. The solid vertical black line indicates the line of no effect (RR = 1), and the dashed vertical black line indicates the overall pooled effect estimate. EVT, endovascular thrombectomy; TNK, tenecteplase; RR, risk ratio; CI, confidence interval.

Good functional outcome (mRS 0–2): Analysis of the full cohort showed a numerically higher rate of good functional outcome with tenecteplase that did not reach statistical significance (RR 1.10, 95% CI 0.97–1.24; *p* = 0.135), with moderate heterogeneity (I-squared = 37.3%, *p* = 0.172) (Figure 4). Subgroup analysis showed a significant benefit within the non-EVT subgroup (RR 1.24, 95% CI 1.03–1.49), but not in the EVT-permitted subgroup (RR 1.05, 95% CI 0.90–1.22; *p* = 0.172). Although the point estimate was higher in the non-EVT subgroup, the interaction test was not statistically significant. Therefore, this result should be interpreted as a possible subgroup pattern rather than as definitive evidence that the treatment effect differs according to EVT availability. The diagnostic plots for these functional results are summarized in Supplementary Figures S6–S10 (Block A).

### 3.4. Revascularization and Early Recovery

**Figure 5:**
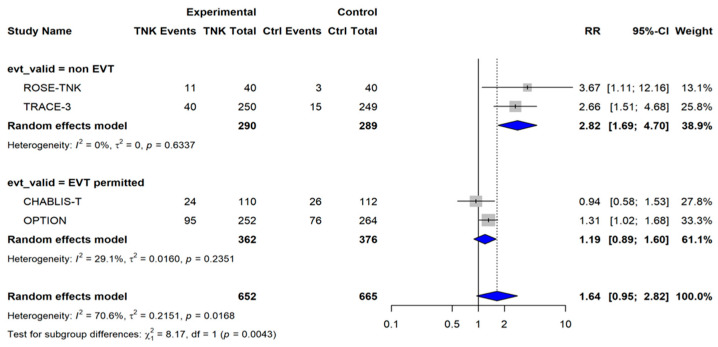
Forest plot of successful recanalization stratified by EVT availability. Gray squares represent study-specific risk ratios (RRs), with square size proportional to study weight; horizontal black lines indicate 95% confidence intervals (CIs). Blue diamonds represent pooled random-effects estimates for each subgroup and the overall analysis, with diamond width indicating the 95% CI. The solid vertical black line indicates the line of no effect (RR = 1), and the dashed vertical black line indicates the overall pooled effect estimate. The effect modification by EVT availability was significant (*p* = 0.004), and overall heterogeneity was substantial (I^2^ = 70.6%). EVT, endovascular thrombectomy; TNK, tenecteplase; RR, risk ratio; CI, confidence interval.

Successful recanalization: Across four studies (*n* = 1317), the overall recanalization rate was numerically higher with tenecteplase but was not statistically significant (RR 1.64, 95% CI 0.95–2.82; *p* = 0.075), with substantial heterogeneity (I-squared = 70.6%) (Figure 5). A significant interaction was observed according to EVT status (*p* = 0.004), with benefits primarily observed in the non-EVT subgroup (RR 2.82, 95% CI 1.69–4.70). This recanalization effect was larger than the corresponding non-EVT estimate for good functional outcome (RR 1.24), indicating that the magnitude of vessel-opening benefit did not translate proportionally into 90-day mRS 0–2 improvement. Sensitivity analyses for recanalization are shown in Supplementary Figures S11–S15 (Block B).

Because recanalization is an intermediate angiographic endpoint, these results were not interpreted as being equivalent to clinical recovery. The divergence between recanalization and functional outcomes was considered particularly relevant when comparing non-EVT and EVT-permitted pathways.

**Figure 6:**
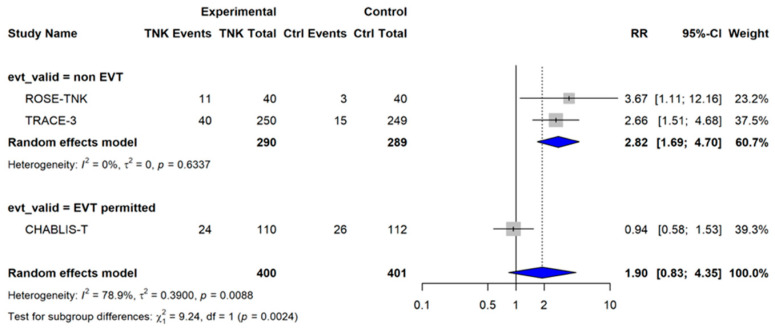
Forest plot of early neurological improvement stratified by EVT availability. Gray squares represent study-specific risk ratios (RRs), with square size proportional to study weight; horizontal black lines indicate 95% confidence intervals (CIs). Blue diamonds represent pooled random-effects estimates for each subgroup and the overall analysis, with diamond width indicating the 95% CI. The solid vertical black line indicates the line of no effect (RR = 1), and the dashed vertical black line indicates the overall pooled effect estimate. The observed benefit was concentrated in non-EVT settings, with a significant subgroup interaction by EVT availability (p = 0.002). EVT, endovascular thrombectomy; TNK, tenecteplase; RR, risk ratio; CI, confidence interval.

Early Neurological Improvement (ENI): In an analysis of three studies (*n* = 801), there was no significant overall difference in ENI (RR 1.90, 95% CI 0.83–4.35; *p* = 0.127), with substantial heterogeneity (I-squared = 78.9%) (Figure 6). However, a significant subgroup interaction was found (*p*-interaction = 0.002), with tenecteplase demonstrating a benefit exclusively in the non-EVT setting (RR 2.82). The high heterogeneity and limited number of contributing studies indicate that ENI findings should be interpreted cautiously despite the significant subgroup interaction. The diagnostics for the ENI results are shown in Supplementary Figures S16–S20 (Block B). A technical summary of these efficacy signals is provided in Supplementary Table S3.

### 3.5. Safety Outcomes

**Figure 7:**
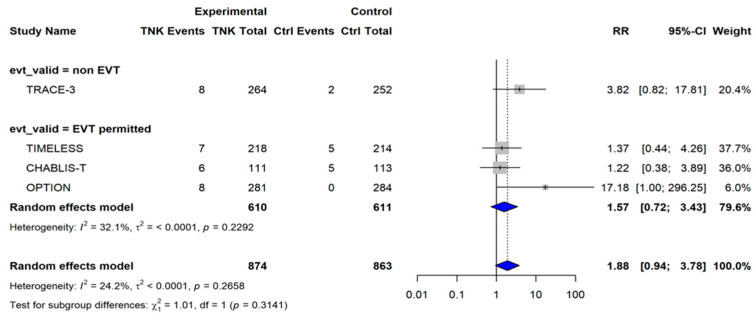
Forest plot of symptomatic intracranial hemorrhage stratified by EVT availability. Gray squares represent study-specific risk ratios (RRs), with square size proportional to study weight; horizontal black lines indicate 95% confidence intervals (CIs). Blue diamonds represent pooled random-effects estimates for each subgroup and the overall analysis, with diamond width indicating the 95% CI. The solid vertical black line indicates the line of no effect (RR = 1), and the dashed vertical black line indicates the overall pooled effect estimate. EVT, endovascular thrombectomy; TNK, tenecteplase; RR, risk ratio; CI, confidence interval.

Symptomatic intracranial hemorrhage (sICH): In the four estimable studies included in the RR-based forest plot (*n* = 1737), sICH occurred in 29/874 patients (3.32%) who received tenecteplase and 12/863 patients (1.39%) in the control group. Tenecteplase showed a nonsignificant trend toward a higher sICH risk (RR 1.88, 95% CI 0.94–3.78; *p* = 0.074), with low-to-moderate heterogeneity (I^2^ = 24.2%) (Figure 7). ROSE-TNK was not displayed in the forest plot because zero sICH events occurred in both treatment arms. Although this result did not reach statistical significance, the direction and width of the confidence interval indicated that a clinically relevant increase in sICH risk could not be excluded. Robustness analyses for sICH are presented in Supplementary Figures S21–S25 (Block C).

**Figure 8:**
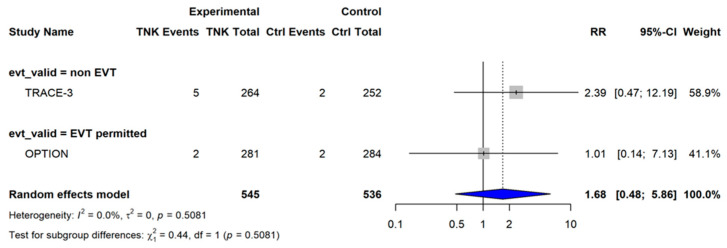
Forest plot of 90-day mortality stratified by EVT availability. Gray squares represent study-specific risk ratios (RRs), with square size proportional to study weight; horizontal black lines indicate 95% confidence intervals (CIs). Blue diamonds represent pooled random-effects estimates, with diamond width indicating the 95% CI. The solid vertical black line indicates the line of no effect (RR = 1), and the dashed vertical black line indicates the overall pooled effect estimate. EVT, endovascular thrombectomy; TNK, tenecteplase; RR, risk ratio; CI, confidence interval.

Moderate or severe systemic bleeding: Analysis of two studies (*n* = 1081) showed no significant difference between the groups (RR 1.68, 95% CI 0.48–5.86; *p* = 0.41; I-squared = 0%) (Figure 8). Because only two studies contributed to this outcome, the estimate remained imprecise despite the absence of statistical heterogeneity. The related diagnostics are shown in Supplementary Figures S26–S30 (Block C).

**Figure 9:**
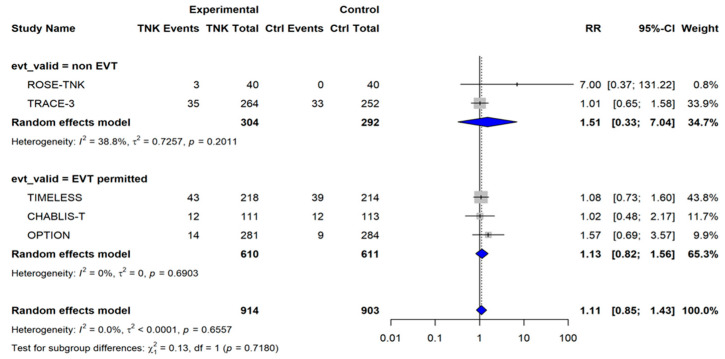
Forest plot of any intracranial hemorrhage stratified by EVT availability. Gray squares represent study-specific risk ratios (RRs), with square size proportional to study weight; horizontal black lines indicate 95% confidence intervals (CIs). Blue diamonds represent pooled random-effects estimates for each subgroup and the overall analysis, with diamond width indicating the 95% CI. The solid vertical black line indicates the line of no effect (RR = 1), and the dashed vertical black line indicates the overall pooled effect estimate. EVT, endovascular thrombectomy; TNK, tenecteplase; RR, risk ratio; CI, confidence interval.

90-Day All-Cause Mortality: Pooled results showed a neutral effect on 90-day survival (RR 1.11, 95% CI 0.85–1.43; *p* = 0.43; I-squared = 0%) (Figure 9). The diagnostics for mortality results are presented in Supplementary Figures S31–S35 (Block C). The confidence interval was compatible with both modest harm and modest benefit; therefore, this finding should be interpreted as no clear mortality difference rather than evidence of identical mortality risk. The diagnostics for mortality results are presented in Supplementary Figures S31–S35 (Block C).

The overall certainty of evidence for each outcome was evaluated using the GRADE approach (Supplementary Table S4), ranging from “high” for functional outcomes to “very low” for early neurological improvements. Certainty was lower for outcomes with substantial heterogeneity, limited contributing studies, or imprecise confidence intervals, particularly for early neurological improvement and safety endpoints.

## 4. Discussion

### 4.1. Principal Findings

This meta-analysis of five RCTs (*n* = 1844) examined whether the clinical benefit of tenecteplase (TNK) in the extended window was observed across different endovascular thrombectomy (EVT) care pathways. The results showed a dichotomy between the functional and mechanistic outcomes. Tenecteplase improved excellent functional outcomes (mRS 0–1 at 90 days: RR 1.25, 95% CI 1.10–1.42; *p* = 0.0005) across the entire cohort with no meaningful heterogeneity (I^2^ = 0%) and no effect modification by EVT status (interaction *p* = 0.96). Tenecteplase improved excellent functional outcomes across the entire cohort; however, a pooled number needed to treat was not calculated because the present analysis pooled relative risks rather than absolute risk differences. However, for intermediate markers of recanalization and early neurological improvement (ENI), EVT status acted as a meaningful treatment modifier with significant interactions (*p* = 0.004 and *p* = 0.002, respectively). Safety signals were largely neutral, although the symptomatic intracranial hemorrhage (sICH) rate trended higher (RR 1.88, *p* = 0.07).

Therefore, the key clinical message is not that TNK works uniformly in all late-window patients but that its apparent benefit depends on the outcome being measured and the care pathway in which it is used. Functional recovery, angiographic vessel opening, and early neurological improvement should not be considered interchangeable endpoints.

This distinction is especially important because the interaction test for good functional outcomes was not statistically significant. A non-significant interaction does not prove that TNK has the same effect on EVT and non-EVT pathways; it may also reflect limited power, few included trials, and wide confidence intervals.

### 4.2. EVT Availability as a Treatment Modifier

The significant recanalization interaction reflects the different roles of TNK across care systems. In the non-EVT subgroup (ROSE-TNK [9] and TRACE-III [11]), pooled recanalization favored TNK substantially (RR 2.82, 95% CI 1.69–4.70), indicating that, where mechanical intervention is unavailable, TNK’s pharmacological activity is the primary determinant of vessel patency. This pattern was consistent across the large vessel occlusion population of TRACE-III [11]. OPTION [13], although categorized as EVT-permitted according to protocol, had limited actual EVT utilization and may similarly reflect a predominantly pharmacological reperfusion pathway.

This helps explain why EVT availability should be viewed as a biologically and operationally important modifier and not merely as a trial design feature. In EVT-eligible patients, TNK is often used as bridging therapy before mechanical reperfusion. In non-EVT patients, TNK may be the main or only active reperfusion treatment. These two clinical contexts can reasonably produce different magnitudes of effects.

Within EVT-permitted settings, mechanical thrombectomy achieves high rates of successful reperfusion [25], a benchmark that extends to 24 h under imaging-based selection [7]. Therefore, TNK functions as a bridging agent rather than a primary revascularization strategy in these settings. Although preprocedural TNK has shown benefits when administered before thrombectomy in acute stroke pathways [26], this signal is more difficult to isolate in an extended window. Trials such as TIMELESS [10], in which EVT was used in most patients, may therefore be underpowered to detect a pharmacological effect that is partly displaced by competing mechanical mechanisms.

Conversely, in settings where EVT is unavailable, delayed, or rarely used, the relative contribution of pharmacological thrombolysis increases. This is clinically relevant for non-thrombectomy centers, transfer-dependent systems, and resource-limited settings, where late-window EVT access remains inconsistent.

### 4.3. Functional Outcomes: Synthesizing the mRS 0–1 and 0–2 Divergence

The mRS 0–1 benefit was the most consistent signal in this analysis, showing stability across trials, regardless of EVT utilization, imaging modality, or stroke type. The mRS 0–2 result was not significant overall (RR 1.10; *p* = 0.13) and was more heterogeneous, largely attributable to CHABLIS-T II [12], in which high mechanical revascularization rates may have compressed the mRS 0–2 distribution and limited the detectable pharmacological additive effect.

The mRS 0–2 subgroup pattern should be interpreted with caution. The non-EVT subgroup showed a significant estimate of good functional outcomes, whereas the EVT-permitted subgroup did not; however, the subgroup interaction was not statistically significant. Therefore, this finding is better framed as a possible care pathway signal rather than definitive proof of different functional efficacy.

The dissociation between the absence of EVT interaction for mRS 0–1 and the clear EVT interaction for recanalization is plausible. Excellent recovery may reflect the effects of TNK beyond macrovascular reopening alone, including downstream microvascular and tissue-level reperfusion biology [27]. Prior meta-analyses that pooled the extended-window population without EVT stratification [6,28,29,30] lacked a framework to detect these care-pathway-specific contributions to the outcome.

Simultaneously, any claim regarding microvascular circulation or tissue biology should be approached with caution. The included trials primarily measured clinical and angiographic outcomes and were not designed to directly quantify microvascular reperfusion, no-reflow, infarct growth, or tissue-level salvage. Therefore, the microvascular benefit remains a plausible explanation rather than a proven mechanism in this dataset.

Baseline differences across trials may also contribute to functional outcome patterns. As shown in Table 1, the included studies differed in terms of age, occlusion status, NIHSS profile, imaging paradigm, and EVT utilization. These differences may affect the probability that vessel opening translates to functional independence.

### 4.4. Recanalization and Early Recovery as Intermediate Signals

The non-significant overall estimates for recanalization (RR 1.64; *p* = 0.07) and ENI (RR 1.90; *p* = 0.13) were structurally driven by care pathway differences. In non-EVT settings, these outcomes showed large and significant benefits (RR 2.82), confirming TNK as a potent primary revascularization tool in these contexts. Recanalization appears to serve as a direct proxy for TNK’s pharmacological activity, whereas functional outcomes reflect a broader downstream recovery. This pattern, established in Phase III trials such as AcT [4] and TRACE-2 [5], now extends into the 4.5–24-h window and supports prespecifying EVT status in future trial designs.

However, vessel opening is a surrogate endpoint and should not be equated with clinical recovery. In the non-EVT subgroup, the effect estimate for recanalization was substantially larger than that for good functional outcomes, suggesting a clinical–radiological mismatch. This may occur when proximal vessel recanalization does not restore adequate tissue-level perfusion or when irreversible infarction occurs before treatment.

Several mechanisms may explain this discrepancy. Recanalization can be incomplete, delayed, or unstable. Distal embolization, impaired collateral flow, microvascular obstruction, and the no-reflow phenomenon may limit tissue reperfusion despite angiographic vessel opening. Reperfusion injury may also contribute to edema, hemorrhagic transformation, and secondary tissue damage. Thus, TNK-related vessel opening may be necessary but insufficient for functional recovery. These findings support the separation of mechanistic endpoints, such as recanalization and ENI, from patient-centered endpoints, such as the 90-day mRS. Future studies should report both categories and ideally include imaging markers of infarct growth, reperfusion quality, collateral status, and hemorrhagic transformation to clarify how early vessel effects translate into disability outcomes.

### 4.5. Tenecteplase Versus Alteplase in the Extended Window

These findings sit within the broader shift toward imaging-guided thrombolysis established by WAKE-UP [14] and EXTEND [15] for alteplase administration. An individual patient data meta-analysis confirmed that imaging-guided alteplase improves functional independence in the late window [16]. More recent evidence from the HOPE [31] and EXPECTS [32] studies further supports the 4.5–24-h period as a viable window for pharmacological reperfusion in selected patients. TNK’s higher fibrin specificity and single-bolus delivery make it a biologically plausible alternative, although its superiority over alteplase cannot be inferred without head-to-head extended-window comparisons. Editorial commentary has framed these late-window data as a meaningful step toward the broader expansion of imaging-guided reperfusion beyond conventional timelines [33].

The practical advantages of TNK therapy are noteworthy. Single-bolus administration can simplify emergency treatment, reduce infusion-related workflow complexity, and facilitate transfer from primary stroke to thrombectomy-capable centers. These advantages are particularly relevant in late-window care, where imaging selection, referral decisions, and transfer logistics create delays.

The potential disadvantages of this approach should also be acknowledged. TNK may increase the bleeding risk in some settings, optimal dosing may differ across stroke populations, and its benefit may depend on the occlusion site, clot burden, collateral status, imaging profile, and whether EVT is available. Therefore, the present findings support TNK as a promising option, but not as a universal replacement for alteplase or EVT.

Reliable frequency-of-use comparisons across countries and stroke systems were not uniformly available in the included trials. Therefore, this review cannot determine how commonly TNK is used in routine late-window practice compared to alteplase or EVT. This should be considered an implementation question for registry-based studies.

### 4.6. Imaging-Guided Selection: A Biological Prerequisite

The neutral outcome of the TWIST trial [34], which used non-contrast CT for patient selection, contrasts with the more favorable signals seen in perfusion- or mismatch-selected populations, such as TRACE-III [11], CHABLIS-T II [12], and OPTION [13]. This suggests that advanced imaging is not simply a preference but may be central to identifying salvageable tissue while controlling the hemorrhagic risk. Data from the NOR-TEST 2 [35] indicate that higher TNK dosing increases the risk of intracranial hemorrhage without corresponding clinical gain. Emerging automated DWI-FLAIR assessment tools may improve decision support and inter-reader agreement in imaging-based selection workflows [36], although real-world implementation still requires calibration and clinical oversight.

Imaging selection may also help explain the gap between recanalization and clinical recovery rates. CT angiography can define the occlusion site and clot burden, CT or MR perfusion can estimate the ischemic core and penumbra, and collateral assessment can help predict whether the tissue remains salvageable. In an extended window, these features may be more clinically informative than time alone.

Newer imaging approaches may further refine patient selection, including automated perfusion core-mismatch quantification, collateral scoring, thrombus imaging, infarct growth estimation, and MRI-based tissue viability assessment. However, these tools should not be considered substitutes for clinical judgment because thresholds vary across platforms, scanners, and health systems.

For future late-window TNK trials, standardized reporting of imaging criteria, infarct core volume, mismatch thresholds, occlusion site, collateral status, and reperfusion quality would improve interpretability and allow clearer comparisons across EVT and non-EVT pathways.

### 4.7. Safety Profile and Mortality Uncertainty

The sICH rate was higher with TNK than with the control (*p* = 0.07). A recent meta-analysis of late-window thrombolysis trials without planned mechanical thrombectomy reported significantly higher odds of sICH without a clear increase in mortality [37]. Our results follow a similar pattern: a modest increase in bleeding risk that does not clearly translate into excess 90-day death, consistent with broader standard-window thrombolysis evidence comparing TNK and alteplase [38]. Nevertheless, the wide mortality confidence interval indicates that a clinically significant increase cannot be excluded.

Therefore, the safety findings should be interpreted cautiously. The absence of a statistically significant increase in mortality does not prove that the mortality risk is identical between groups, and the non-significant sICH trend may still be clinically important. This is especially relevant in extended-window treatment, where infarct core size, blood–brain barrier injury, reperfusion injury, and imaging-selection accuracy may influence hemorrhagic risk.

Because systemic bleeding was reported in only two studies, confidence in this estimate is limited. Future trials should standardize bleeding definitions and report symptomatic intracranial hemorrhage, intracranial hemorrhage, systemic bleeding, and fatal bleeding separately.

### 4.8. Global Health Equity and Resource-Limited Settings

The EVT interaction is particularly important for patients in settings with limited thrombectomy access [8]. For these populations, TNK may be the only practical reperfusion option. Its single-bolus administration also offers workflow advantages in drip-and-ship systems, and structured transfer redesign has demonstrated that meaningful reductions in door-in-door-out time are achievable when thrombolysis and transfer activation are parallelized [39]. Expanding access to advanced imaging remains necessary to safely operationalize late-window reperfusion at scale.

Generalizability remains a key concern in this study. Four of the five included trials were conducted in China, whereas only TIMELESS was conducted in the United States and Canada. Regional differences in stroke referral pathways, EVT access, prehospital triage, imaging availability, treatment delays, rehabilitation access, and background population characteristics may influence both treatment delivery and outcome.

Potential biological differences in the fibrinolytic response have been proposed in the broader thrombolysis literature; however, the present trial set cannot directly test whether genetic, racial, or ethnic factors modify the TNK response. Therefore, these issues should be framed as possible sources of external validity uncertainty, not as established explanations for the observed findings.

The strongest global health implication is practical: TNK may be most valuable where EVT access is limited, but safe implementation still requires imaging capacity, hemorrhage risk assessment, trained stroke teams, and reliable post-treatment monitoring.

### 4.9. Strengths and Limitations

The primary strength of this analysis is the EVT-stratified framework and inclusion of the most recent randomized evidence, making this a current synthesis of available data. The Hartung–Knapp adjustment was applied throughout to maintain conservative estimates appropriate for the limited number of included trials [22].

The limitations of this study include the small number of included studies (*n* = 5) and the structural differences between the trials in the non-EVT subgroup. The geographic concentration of data in China limits the generalizability of this study to other health systems in other countries. In addition, the 90-day mortality confidence interval was too wide to confirm a definitive neutral survival effect.

Several additional limitations should be considered. First, subgroup interaction tests were based on a few trials and may have been underpowered; therefore, non-significant interaction findings, particularly for mRS 0–2, should not be interpreted as proof of equivalent treatment effects. Second, EVT classification was based on trial protocol permissibility rather than uniform actual EVT use, which may have misclassified some care pathways. Third, baseline differences in age, occlusion type, NIHSS score, imaging strategy, and EVT utilization may have contributed to the between-study heterogeneity.

Fourth, recanalization and ENI are intermediate endpoints and cannot fully substitute for patient-centered functional outcomes. Fifth, the included studies differed in their imaging-selection criteria and outcome definitions, limiting direct comparability. Sixth, the small number of studies restricts the reliability of funnel plot assessment and limits the detection of small-study effects or publication bias. Finally, because most trials were conducted in one country, the findings require confirmation in more diverse health systems and populations in the future.

### 4.10. Future Directions

Future trials should prioritize head-to-head comparisons of TNK and alteplase in the extended window and prospectively stratify EVT availability, particularly in low-income settings. Evidence gaps remain for mild stroke (NIHSS < 6) and posterior circulation AIS within an extended window.

Future studies should also separate EVT-eligible, EVT-treated, EVT-delayed, and non-EVT populations rather than grouping them only by broad trial protocols. Trials should report absolute risks alongside relative effects, allowing the estimation of the number of patients needed to treat and the number of patients needed to harm.

Imaging protocols should be standardized, including core volume, mismatch criteria, occlusion sites, collateral status, and reperfusion quality. Future studies should also test whether early recanalization produces durable tissue reperfusion and whether no-reflow or reperfusion injury explains the gap between angiographic and clinical outcomes.

Large pragmatic registries may be useful for estimating the real-world frequency of TNK use, workflow benefits, geographic variations, and implementation safety in systems with limited EVT access.

## 5. Conclusions

In imaging-selected patients with acute ischemic stroke presenting 4.5–24 h after onset, EVT availability appears to modify the effect of tenecteplase on mechanistic outcomes. Tenecteplase improved excellent functional outcomes at 90 days (mRS 0–1; RR 1.25, 95% CI 1.10–1.42) across care pathways, whereas its benefits for recanalization and early neurological improvement were largely confined to non-EVT settings (*p*-interaction ≤ 0.004). Safety outcomes were broadly neutral, with no clear effect on mortality and a non-significant trend toward more symptomatic intracranial hemorrhage under imaging-guided selection. For patients without access to endovascular therapy, tenecteplase is a practical and biologically plausible reperfusion option. Future extended-window trials should prespecify EVT status as a key stratification factor.

These findings should be interpreted as supportive but not definitive. The evidence suggests that TNK may be most clinically relevant when pharmacological reperfusion is the main available treatment pathway; however, the gap between vessel opening and functional recovery, the limited number of trials, and the geographic concentration of evidence require cautious interpretation.

## Figures and Tables

**Table 1:** Characteristics of Included Studies.

Study	Phase	Country	Period	Inclusion Criteria	Randomized Groups, *n*	Age, y (Mean/Median)	Male Sex, %	NIHSS (Mean/Median)	EVT Utilization, %
ROSE-TNK (2023) [9]	II	China	2021–2022	NIHSS 6–25; DWI-FLAIR mismatch	TNK, 40; Control, 40	TNK, 62.7; Control, 62.8	TNK, 77.5; Control, 65.0	TNK, 7.5; Control, 7.0	0
TIMELESS (2024) [10]	III	USA/Canada	2019–2022	NIHSS 5; LVO; perfusion screening	TNK, 228; Placebo, 230	TNK, 72; Placebo, 73	TNK, 46.5; Placebo, 46.5	TNK, 12; Placebo, 12	77.3
TRACE-III (2024) [11]	III	China	2022–2023	NIHSS 6–25; LVO; perfusion screening	TNK, 264; Control, 252	TNK, 67; Control, 68	TNK, 69.3; Control, 66.3	TNK, 11; Control, 10	0 *
CHABLIS-T II (2025) [12]	IIb	China	2021–2023	LVO (ICA/MCA/ACA); CTP screening	TNK, 111; Control, 113	TNK, 64.2; Control, 63.6	TNK, 72.1; Control, 70.8	TNK, 9; Control, 9	54.9
OPTION (2026) [13]	III	China	2023–2025	NIHSS 6–25; non-LVO; CTP screening	TNK, 282; Control, 284	TNK, 69; Control, 67	TNK, 62.4; Control, 68.3	TNK, 7; Control, 6	1.3

* None before randomization; rescue EVT was performed in four patients in the TNK group and five patients in the control group.

**Table 2:** Summary of Primary Efficacy and Safety Outcomes Stratified by EVT Availability.

Outcome	Overall RR (95% CI)	Overall *p*-Value	Non-EVT Subgroup RR (95% CI)	EVT-Permitted Subgroup RR (95% CI)	*p*-Interaction	Heterogeneity (I^2^)
Efficacy	Overall RR (95% CI)	Overall *p*-value	Non-EVT Subgroup RR (95% CI)	EVT-Permitted Subgroup RR (95% CI)	*p*-interaction	Heterogeneity (I^2^)
Excellent Functional Outcome (mRS 0–1)	1.25 (1.10–1.42)	<0.001	1.26 (1.00–1.59)	1.25 (1.07–1.46)	0.961	0%
Good Functional Outcome (mRS 0–2)	1.10 (0.97–1.24)	0.135	1.24 (1.03–1.49)	1.05 (0.90–1.22)	0.172	37.3%
Successful Recanalization	1.64 (0.95–2.82)	0.075	2.82 (1.69–4.70)	1.19 (0.89–1.60)	0.004	70.6%
Early Neurological Improvement	1.90 (0.83–4.35)	0.127	2.82 (1.69–4.70)	0.94 (0.58–1.53)	0.002	78.9%
Safety						
Symptomatic Intracranial Hemorrhage	1.88 (0.94–3.78)	0.074	3.82 (0.82–17.81)	1.57 (0.72–3.43)	0.314	24.2%
90-Day All-Cause Mortality	1.11 (0.85–1.43)	0.43	1.51 (0.33–7.04)	1.16 (0.82–1.65)	0.72	0%

Notes: RR, risk ratio; CI, confidence interval; mRS, modified Rankin Scale; EVT, endovascular thrombectomy. Subgroup *p*-interaction values assessed statistical evidence of differential treatment effects across EVT-defined settings; non-significant interaction tests were not interpreted as proof of equivalent treatment effects.

## Data Availability

No new data were created or analyzed during this study. Data sharing is not applicable to this study. All analyzed data are presented in the Supplementary Tables.

## Supplementary Materials

The following supporting information can be downloaded at: https://www.mdpi.com/article/10.3390/healthcare14111470/s1, Supplementary Table S1: The PRISMA 2020 checklist, detailing the location of all items reported in the systematic review and meta-analysis. Supplementary Table S2: Detailed search strings and results across PubMed, Embase, Scopus, and the Cochrane Library for searches conducted through February 2026. Supplementary Table S3: A summary of efficacy and safety outcomes, providing definitions and statistical summaries (risk ratios, *p*-values, and heterogeneity) for functional outcomes, recanalization, early neurological improvement (ENI), symptomatic intracranial hemorrhage (sICH), and mortality. Supplementary Table S4: GRADE Assessment of Outcomes, evaluating the certainty of evidence (High to Very Low) and importance for each primary and secondary outcome. Supplementary Figures S1–S5: Excellent Functional Outcome (mRS 0–1); Figure S1: Sensitivity analysis demonstrating the robustness of the pooled effect estimate; Figures S2–S5: Heterogeneity and publication bias diagnostics (GOSH, Baujat, Drapery, and Funnel plots). Supplementary Figures S6–S10: Good Functional Outcome (mRS 0–2); Figure S6: Sensitivity analysis showing the stability of findings. Figures S7–S10: Subset heterogeneity and publication bias assessment plots. Supplementary Figures S11–S15: Recanalization diagnostics and sensitivities. Supplementary Figures S16–S20: Early Neurological Improvement (ENI) diagnostics and sensitivity. Supplementary Figures S21–S25: Symptomatic Intracranial Hemorrhage (sICH) diagnostics and sensitivity. Supplementary Figures S26–S30: Moderate-to-Severe Systemic Bleeding diagnostics and sensitivity. Supplementary Figures S31–S35: c90-Day All-Cause Mortality diagnostics and sensitivity.

## Abbreviations

The following abbreviations are used in this manuscript:

AIS (acute ischemic stroke), TNK (tenecteplase), rtPA (recombinant tissue plasminogen activator), and EVT (endovascular thrombectomy). Clinical assessments and outcomes are denoted by mRS (modified Rankin Scale), NIHSS (National Institutes of Health Stroke Scale), sICH (symptomatic intracranial hemorrhage), ENI (early neurological improvement), and TICI (thrombolysis in cerebral infarction). Pathophysiological and biochemical terms include LVO, large vessel occlusion; BBB, blood–brain barrier; and PAI-1, plasminogen activator inhibitor-1. Imaging modalities are abbreviated as CT (computed tomography), CTP (computed tomography perfusion), MRI (magnetic resonance imaging), DWI (diffusion-weighted imaging), and FLAIR (fluid-attenuated inversion recovery). Methodological and statistical terms: RCT (randomized controlled trial), PRISMA (Preferred Reporting Items for Systematic Reviews and Meta-Analyses), PROSPERO (International Prospective Register of Systematic Reviews), RoB 2 (Cochrane Risk of Bias tool, version 2), and GRADE (Grading of Recommendations Assessment, Development and Evaluation). Statistical metrics are represented by risk ratio (RR), confidence interval (CI), restricted maximum-likelihood (REML), graphical display of study heterogeneity (GOSH), and area under the curve (AUC).
