# Supplementary material for: Tenecteplase in the Extended 4.5–24-Hour Window for Acute Ischemic Stroke: An Updated Meta-Analysis of RCTs with EVT-Stratified Subgroup Analysis
Source: Healthcare (Basel). 2026 May 26;14(11):1470. doi: 10.3390/healthcare14111470 (PMC13257171; doi:10.3390/healthcare14111470)
Supplement: Supplementary file 1 [file healthcare-14-01470-s001.zip › healthcare-4295501-supplementary.pdf]

Tenecteplase in the Extended 4.5–24-Hour Window for Acute Ischemic Stroke: An Updated Meta-analysis of RCTs with EVT-Stratified Subgroup Analysis

Sadia Qazi[1*], Arsalan Ahmed[2], Mazhar Ali[3], Muhammad Usman Iqbal[4], Eshal Atif [5],Zain Ali[6], Abdullah Imtiaz[6], Nabahat Shafi [7], Muhammad Hassan Imtiaz [8], Mohammad Dawar Zahid [9], Muhammad Sharjeel Abbas[10], Muhammad Atif Mazhar [1]

Supplementary Table 1. PRISMA 2020 checklist of items reported in this systematic review and meta-analysis.

Section and Topic	Item #	Checklist item	Location where item is reported
**TITLE**			
Title	1	Identify the report as a systematic review.	1
**ABSTRACT**			
Abstract	2	See the PRISMA 2020 for Abstracts checklist.	2-3
**INTRODUCTION**			
Rationale	3	Describe the rationale for the review in the context of existing knowledge.	3-4
Objectives	4	Provide an explicit statement of the objective(s) or question(s) the review addresses.	3-4
**METHODS**			
Eligibility criteria	5	Specify the inclusion and exclusion criteria for the review and how studies were grouped for the syntheses.	5
Information sources	6	Specify all databases, registers, websites, organizations, reference lists and other sources searched or consulted to identify studies. Specify the date when each source was last searched or consulted.	5
Search strategy	7	Present the full search strategies for all databases, registers and websites, including any filters and limits used.	5

Section and Topic	Item #	Checklist item	Location where item is reported
Selection process	8	Specify the methods used to decide whether a study met the inclusion criteria of the review, including how many reviewers screened each record and each report retrieved, whether they worked independently, and if applicable, details of automation tools used in the process.	5
Data collection process	9	Specify the methods used to collect data from reports, including how many reviewers collected data from each report, whether they worked independently, any processes for obtaining or confirming data from study investigators, and if applicable, details of automation tools used in the process.	5
Data items	10a	List and define all outcomes for which data were sought. Specify whether all results that were compatible with each outcome domain in each study were sought (e.g. for all measures, time points, analyses), and if not, the methods used to decide which results to collect.	5
	10b	List and define all other variables for which data were sought (e.g. participant and intervention characteristics, funding sources). Describe any assumptions made about any missing or unclear information.	5
Study risk of bias assessment	11	Specify the methods used to assess risk of bias in the included studies, including details of the tool(s) used, how many reviewers assessed each study and whether they worked independently, and if applicable, details of automation tools used in the process.	6
Effect measures	12	Specify for each outcome the effect measure(s) (e.g. risk ratio, mean difference) used in the synthesis or presentation of results.	5-6
Synthesis methods	13a	Describe the processes used to decide which studies were eligible for each synthesis (e.g. tabulating the study intervention characteristics and comparing against the planned groups for each synthesis (item #5)).	5
	13b	Describe any methods required to prepare the data for presentation or synthesis, such as handling of missing summary statistics, or data conversions.	5-6

Section and Topic	Item #	Checklist item	Location where item is reported
	13c	Describe any methods used to tabulate or visually display results of individual studies and syntheses.	6
	13d	Describe any methods used to synthesize results and provide a rationale for the choice(s). If meta-analysis was performed, describe the model(s), method(s) to identify the presence and extent of statistical heterogeneity, and software package(s) used.	5-6
	13e	Describe any methods used to explore possible causes of heterogeneity among study results (e.g. subgroup analysis, meta-regression).	6
	13f	Describe any sensitivity analyses conducted to assess robustness of the synthesized results.	6
Reporting bias assessment	14	Describe any methods used to assess risk of bias due to missing results in a synthesis (arising from reporting biases).	7
Certainty assessment	15	Describe any methods used to assess certainty (or confidence) in the body of evidence for an outcome.	6
**RESULTS**			
Study selection	16a	Describe the results of the search and selection process, from the number of records identified in the search to the number of studies included in the review, ideally using a flow diagram.	7
	16b	Cite studies that might appear to meet the inclusion criteria, but which were excluded, and explain why they were excluded.	7
Study characteristics	17	Cite each included study and present its characteristics.	7
Risk of bias in studies	18	Present assessments of risk of bias for each included study.	8
Results of individual studies	19	For all outcomes, present, for each study: (a) summary statistics for each group (where appropriate) and (b) an effect estimate and its precision (e.g. confidence/credible interval), ideally using structured tables or plots.	8-12
Results of syntheses	20a	For each synthesis, briefly summarise the characteristics and risk of bias among contributing studies.	8-12

Section and Topic	Item #	Checklist item	Location where item is reported
	20b	Present results of all statistical syntheses conducted. If meta-analysis was done, present for each the summary estimate and its precision (e.g. confidence/credible interval) and measures of statistical heterogeneity. If comparing groups, describe the direction of the effect.	8-12
	20c	Present results of all investigations of possible causes of heterogeneity among study results.	8-12
	20d	Present results of all sensitivity analyses conducted to assess the robustness of the synthesized results.	8-12
Reporting biases	21	Present assessments of risk of bias due to missing results (arising from reporting biases) for each synthesis assessed.	8-12
Certainty of evidence	22	Present assessments of certainty (or confidence) in the body of evidence for each outcome assessed.	Supplementary Table 4.
**DISCUSSION**			
Discussion	23a	Provide a general interpretation of the results in the context of other evidence.	12
	23b	Discuss any limitations of the evidence included in the review.	14
	23c	Discuss any limitations of the review processes used.	14
	23d	Discuss implications of the results for practice, policy, and future research.	16
**OTHER INFORMATION**			
Registration and protocol	24a	Provide registration information for the review, including register name and registration number, or state that the review was not registered.	7
	24b	Indicate where the review protocol can be accessed, or state that a protocol was not prepared.	7
	24c	Describe and explain any amendments to information provided at registration or in the protocol.	7

Section and Topic	Item #	Checklist item	Location where item is reported
Support	25	Describe sources of financial or non-financial support for the review, and the role of the funders or sponsors in the review.	2
Competing interests	26	Declare any competing interests of review authors.	2
Availability of data, code and other materials	27	Report which of the following are publicly available and where they can be found: template data collection forms; data extracted from included studies; data used for all analyses; analytic code; any other materials used in the review.	2

From: Page MJ, McKenzie JE, Bossuyt PM, Boutron I, Hoffmann TC, Mulrow CD, et al. The PRISMA 2020 statement: an updated guideline for reporting systematic reviews. BMJ 2021;372:n71. doi: 10.1136/bmj.n71. This work is licensed under CC BY 4.0. To view a copy of this license, visit https://creativecommons.org/licenses/by/4.0/

Supplementary Table 2. Detailed search strings and results across medical databases (inception to February 2026).

Database	Search String	No. Of Studies
Pubmed	(tenecteplase[Title/Abstract]<br>OR TNK[Title/Abstract]<br>OR "TNK-tPA"[Title/Abstract]<br>OR TNKase[Title/Abstract]<br>OR Metalyse[Title/Abstract])<br>AND<br>("Stroke"[MeSH Terms]<br>OR "Brain Ischemia"[MeSH Terms]<br>OR "Cerebral Infarction"[MeSH Terms]<br>OR "acute ischemic stroke"[Title/Abstract]<br>OR "acute ischaemic stroke"[Title/Abstract]<br>OR "ischemic stroke"[Title/Abstract]<br>OR "ischaemic stroke"[Title/Abstract]<br>OR "brain ischemia"[Title/Abstract]<br>OR "brain ischaemia"[Title/Abstract]<br>OR "cerebral infarction"[Title/Abstract]<br>OR "ischemic cerebrovascular accident"[Title/Abstract]<br>OR "ischaemic cerebrovascular accident"[Title/Abstract]<br>OR AIS[Title/Abstract])	696
Embase	('tenecteplase':ab,ti<br>OR 'tnk':ab,ti<br>OR 'tnk-tpa':ab,ti<br>OR 'tnkase':ab,ti<br>OR 'metalyse':ab,ti)<br>AND('exp cerebrovascular accident'/exp<br>OR 'exp brain ischemia'/exp<br>OR 'exp brain infarction'/exp<br>OR 'acute ischemic stroke':ab,ti<br>OR 'acute ischaemic stroke':ab,ti	2600

	OR 'ischemic stroke':ab,ti<br>OR 'ischaemic stroke':ab,ti<br>OR 'brain ischemia':ab,ti<br>OR 'brain ischaemia':ab,ti<br>OR 'cerebral infarction':ab,ti<br>OR 'ischemic cerebrovascular accident':ab,ti<br>OR 'ischaemic cerebrovascular accident':ab,ti<br>OR 'ais':ab,ti)	
Scopus	TITLE-ABS-KEY<br>(tenecteplase OR TNK OR "TNK-tPA" OR TNKase OR Metalyse) AND TITLE-ABS-KEY ("acute ischemic stroke" OR "acute ischaemic stroke" OR "ischemic stroke" OR "ischaemic stroke" OR "brain ischemia" OR "brain ischaemia" OR "cerebral infarction" OR "ischemic cerebrovascular accident"<br>OR "ischaemic cerebrovascular accident" OR AIS)	1238
Cochrane	(tenecteplase:ti,ab,kw<br>OR TNK:ti,ab,kw<br>OR "TNK-tPA":ti,ab,kw<br>OR TNKase:ti,ab,kw<br>OR Metalyse:ti,ab,kw)<br>AND([mh "Stroke"]<br>OR [mh "Brain Ischemia"]<br>OR [mh "Cerebral Infarction"]<br>OR "acute ischemic stroke":ti,ab,kw<br>OR "acute ischaemic stroke":ti,ab,kw<br>OR "ischemic stroke":ti,ab,kw<br>OR "ischaemic stroke":ti,ab,kw<br>OR "brain ischemia":ti,ab,kw<br>OR "brain ischaemia":ti,ab,kw<br>OR "cerebral infarction":ti,ab,kw<br>OR "ischemic cerebrovascular accident":ti,ab,kw<br>OR "ischaemic cerebrovascular accident":ti,ab,kw<br>OR AIS:ti,ab,kw)	542

Footnote: Search conducted in PubMed, Embase, Scopus, and the Cochrane Library. Terms include Medical Subject Headings (MeSH) and free-text keywords.

Supplementary Table 3. Summary of efficacy and safety outcomes for tenecteplase in the 4.5–24-hour window.

Outcome	Definition and Statistical Summary
**Excellent Functional Outcome**	Achievement of mRS 0–1 at 90 days showed a significant benefit for the intervention (RR 1.25, p=0.0005) with 0% heterogeneity.
**Good Functional Outcome**	Achievement of mRS 0–2 at 90 days; showed a non-significant numerical improvement (RR 1.10, p=0.1345) with moderate heterogeneity (I2=37.3%).
**Recanalization**	Successful restoration of blood flow in the occluded vessel showed a non-significant overall trend (RR 1.64, p=0.0748) but a significant benefit in non-EVT patients.
**Early Neurological Improvement**	Significant reduction in NIHSS score within 24–72 hours; showed no overall difference (p=0.1266) due to high heterogeneity driven by EVT status.
**sICH**	Symptomatic intracranial hemorrhage within 24–72 hours; showed no significant increase in risk compared to control (RR 1.88, p=0.0737).
**Death within 90 Days**	All-cause mortality reported at 90 days showed no significant difference between groups (RR 1.11, p=0.4171) with no observed heterogeneity (I2=0%).

Footnote: RR = risk ratio; mRS = modified Rankin Scale; sICH = symptomatic intracranial hemorrhage; p-values represent the significance of the pooled effect estimate.

Supplementary Table 4. GRADE Assessment of Outcomes

Certainty assessment							№ of patients		Effect		Certainty	Importance
№ of studies	Study design	Risk of bias	Inconsistency	Indirectness	Imprecision	Other considerations	[Tenecteplase]	[Placebo]	Relative (95% CI)	Absolute (95% CI)		
**Excellent Functional outcome**												
5	Randomized trials	not serious	not serious	not serious	not serious	none	348/923 (37.7%)	275/918 (30.0%)	RR 1.25 (1.10 to 1.42)	75 more per 1,000 (from 30 more to 126 more)	⊕⊕⊕⊕ High	CRITICAL
**Good Functional Outcome**												
5	Randomized trials	not serious	not serious	not serious	not serious	none	478/923 (51.8%)	428/918 (46.6%)	RR 1.10 (0.97 to 1.24)	57 more per 1,000 (from 17 fewer to 137 more)	⊕⊕⊕⊕ High[a]	CRITICAL
**Recanalization**												

Certainty assessment							№ of patients		Effect		Certainty	Importance
№ of studies	Study design	Risk of bias	Inconsistency	Indirectness	Imprecision	Other considerations	[Tenecteplase]	[Placebo]	Relative (95% CI)	Absolute (95% CI)		
4	Randomized trials	not serious	serious[b]	not serious	not serious	none	170/652 (26.1%)	129/665 (19.4%)	RR 1.64 (0.95 to 2.82)	124 more per 1,000 (from 10 fewer to 353 more)	⊕⊕⊕◯ Moderate[b]	CRITICAL
**Early Neurological Improvement**												
3	Randomized trials	not serious	very serious[c]	not serious	serious[d]	none	75/400 (18.8%)	44/401 (11.0%)	RR 1.90 (0.83 to 4.35)	99 more per 1,000 (from 19 fewer to 368 more)	⊕◯◯◯ Very low[c,d]	IMPORTANT
**sICH**												

Certainty assessment							№ of patients		Effect		Certainty	Importance
**№ of studies**	**Study design**	**Risk of bias**	**Inconsistency**	**Indirectness**	**Imprecision**	**Other considerations**	**[Tenecteplase]**	**[Placebo]**	**Relative (95% CI)**	**Absolute (95% CI)**		
4	Randomized trials	not serious	not serious	not serious	serious	none	29/914 (3.2%)	12/903 (1.3%)	RR 1.88 (0.94 to 3.78)	12 more per 1,000 (from 1 fewer to 37 more)	⊕⊕⊕◯ Moderate	CRITICAL
**Mortality within 90 days**												
5	Randomized trials	not serious	not serious	not serious	serious	none	107/914 (11.7%)	93/903 (10.3%)	RR 1.11 (0.85 to 1.43)	11 more per 1,000 (from 15 fewer to 44 more)	⊕⊕⊕◯ Moderate	CRITICAL
**Moderate or severe systemic bleeding within 90-days**												
2	Randomized trials	not serious	not serious	not serious	serious	none	7/545 (1.3%)	4/536 (0.7%)	RR 1.68 (0.48 to 5.86)	5 more per 1,000 (from 4 fewer to 36 more)	⊕⊕⊕◯ Moderate	CRITICAL

CI: confidence interval; **RR:** risk ratio. [a] : No downgrading was applied, [b] : Downgraded one level for inconsistency because treatment effects varied across trials, largely according to EVT pathway, [c] : Downgraded two levels for very serious inconsistency because effect estimates differed markedly across studies and EVT-stratified settings, [d]: Downgraded one level for imprecision because the confidence interval crossed no effect and remained compatible with both meaningful benefit and no clear benefit.

Block A: Functional Outcomes (Figs 1–10)

Supplementary Figs 1–5: Excellent Functional Outcome (mRS 0–1):
Supplementary Figure 1. Leave-One-Out Sensitivity Analysis

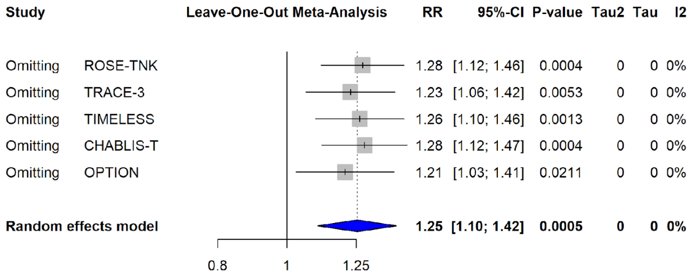

Legend: Leave-one-out sensitivity analysis of excellent functional outcome.
Leave-one-out sensitivity analysis showing the influence of sequential omission of individual studies on the pooled effect estimate for excellent functional outcome, demonstrating the robustness of the overall findings.

Supplementary Figure 2. GOSH Analysis

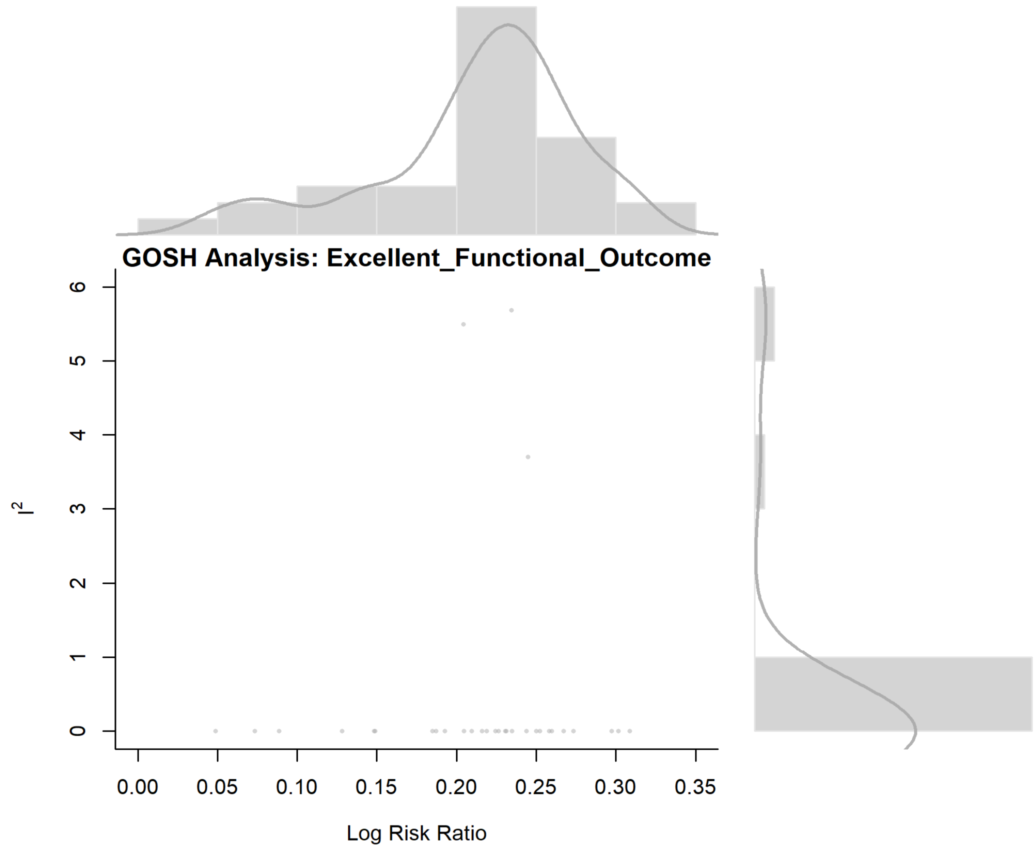

Legend: GOSH plot of excellent functional outcome.
Graphic display of study heterogeneity (GOSH) plot showing the distribution of pooled log risk ratios and heterogeneity estimates across all possible study subsets for excellent functional outcome.

Supplementary Figure 3. Baujat Plot

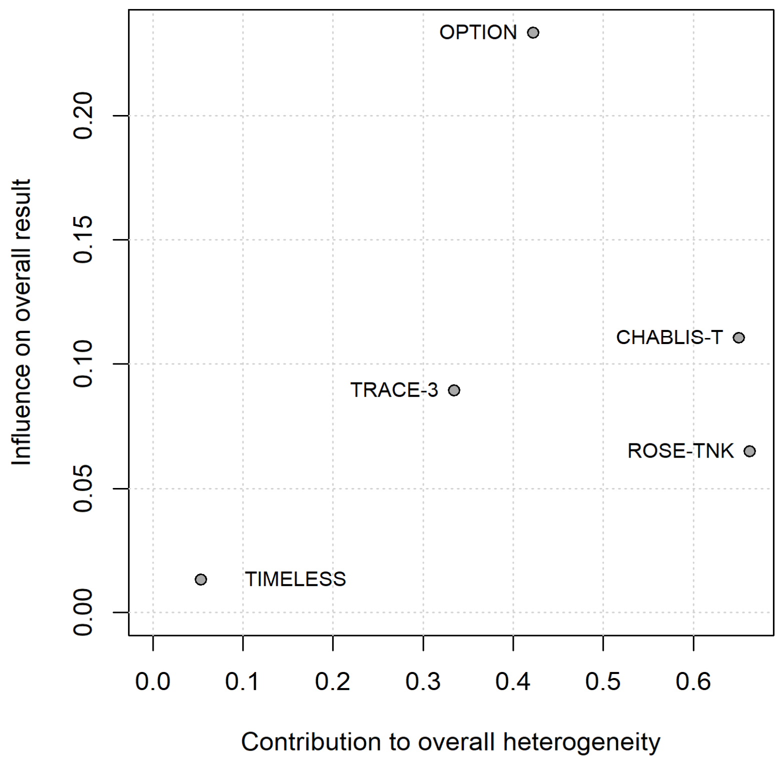

Legend: Baujat plot of excellent functional outcome.
Baujat plot identifying the relative contribution of each included study to overall heterogeneity and its influence on the pooled effect estimate for excellent functional outcome.

Supplementary Figure 4. p-value Functions (Drapery Plot)

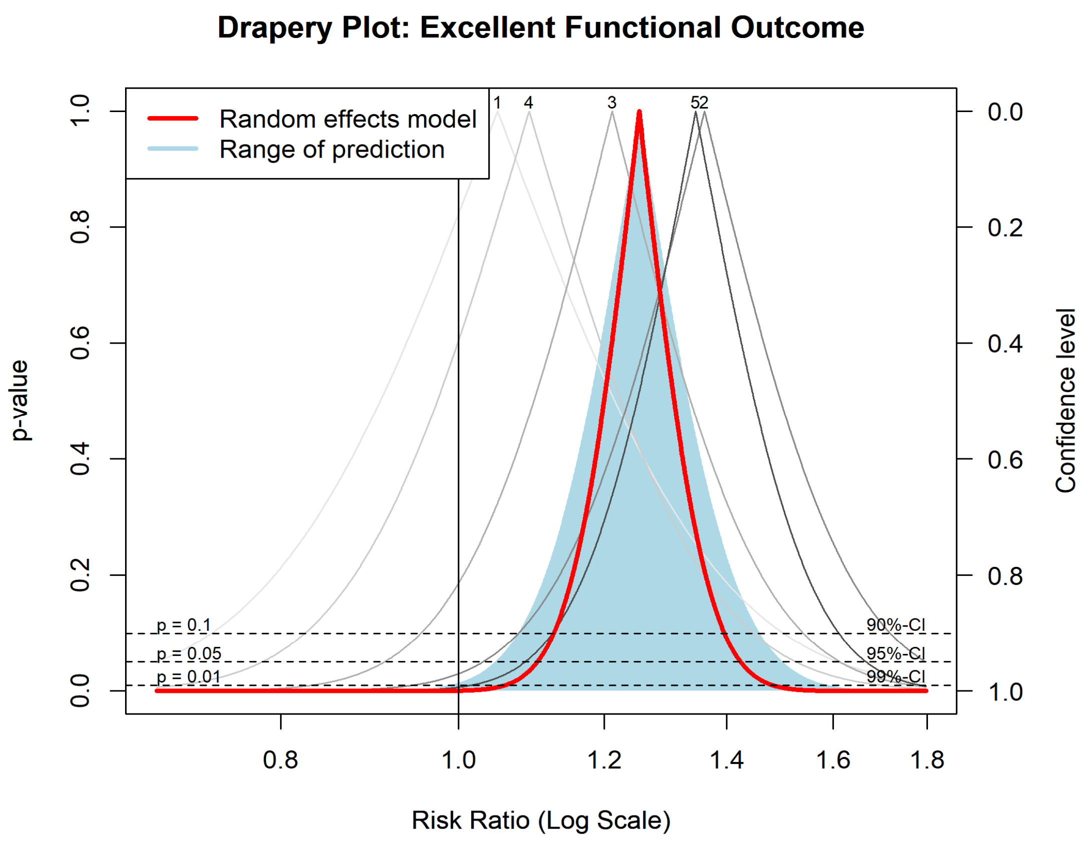

Legend: Drapery plot of excellent functional outcome.
Drapery plot illustrates the p-value functions, confidence levels, pooled random-effects estimate, and prediction range for excellent functional outcome.

Supplementary Figure 5. Funnel Plot

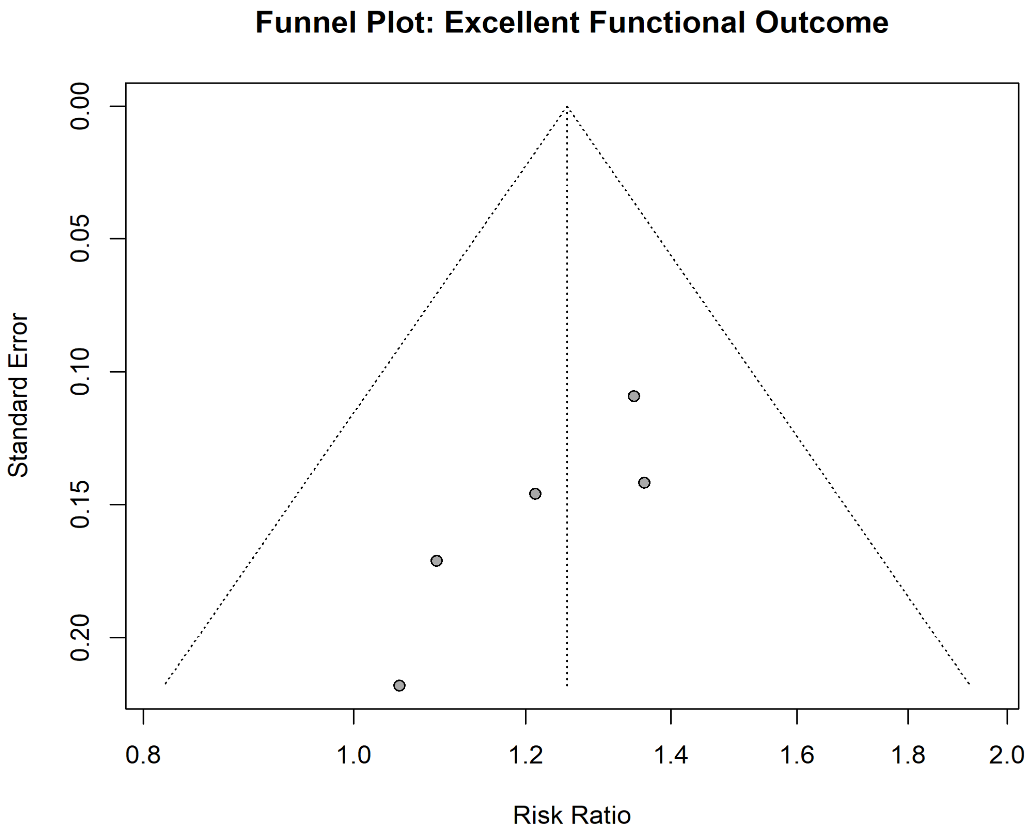

Legend: Funnel plot of excellent functional outcome.
Funnel plot assessing potential small-study effects and publication bias for the outcome of excellent functional outcome across the included studies.

Figs 6–10: Good Functional Outcome (mRS 0–2):

Supplementary Figure 6. Leave-One-Out Sensitivity Analysis

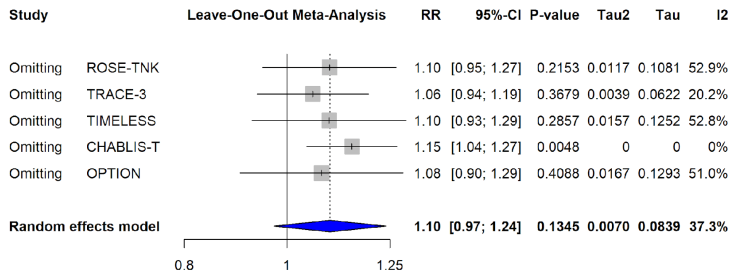

Legend: Leave-one-out sensitivity analysis of good functional outcome.
Leave-one-out sensitivity analysis showing the influence of sequential omission of individual studies on the pooled effect estimate for good functional outcome and demonstrating the stability of the overall findings.

Supplementary Figure 7. GOSH Analysis

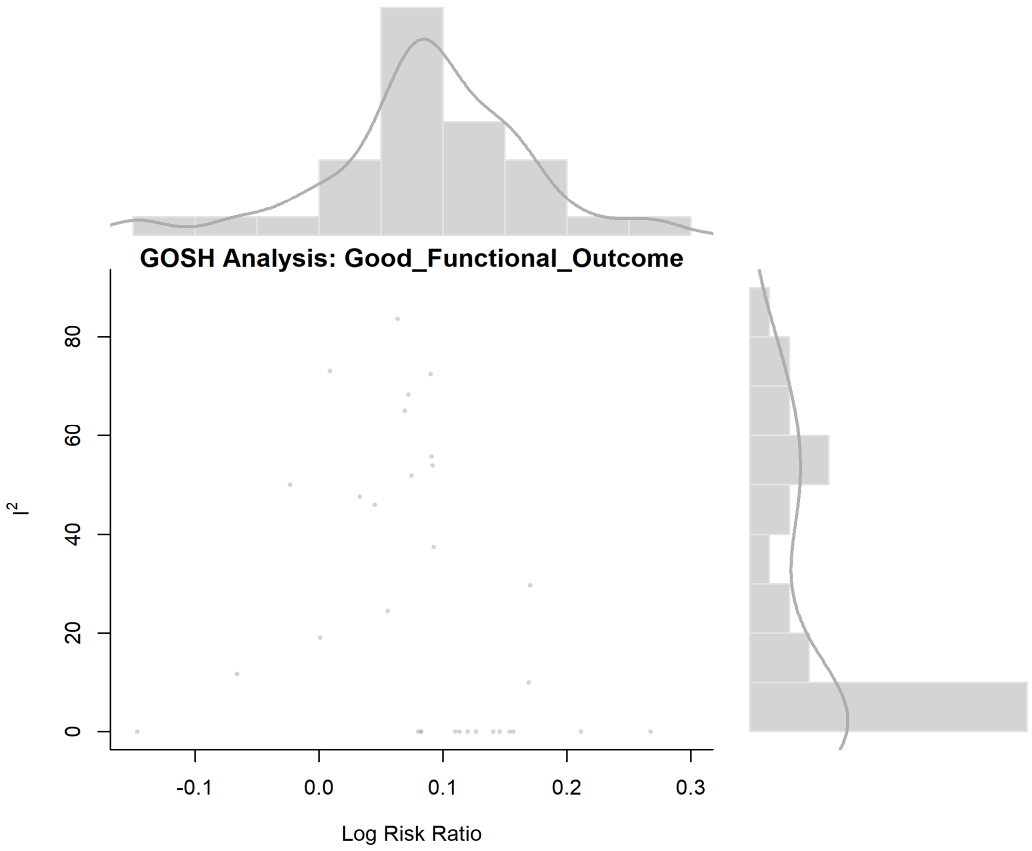

Legend: GOSH plot of good functional outcome.
Graphic display of study heterogeneity (GOSH) plot showing the distribution of pooled log risk ratios and heterogeneity estimates across all possible study subsets for good functional outcome.

Supplementary Figure 8. Baujat Plot

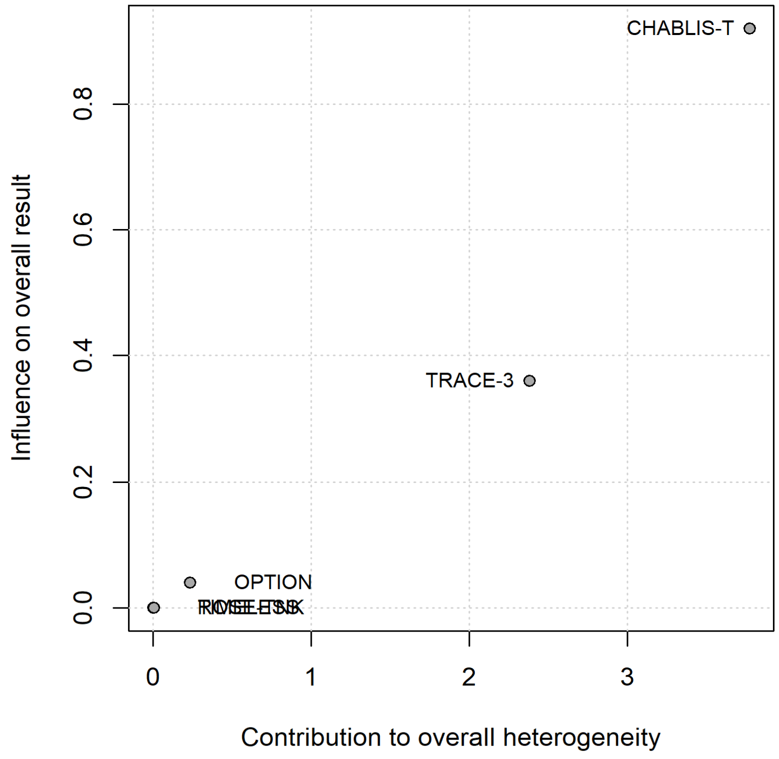

Legend: Baujat plot of good functional outcome.

Baujat plot identifying the relative contribution of each included study to overall heterogeneity and its influence on the pooled effect estimate for good functional outcome.

Supplementary Figure 9. p-value Functions (Drapery Plot)

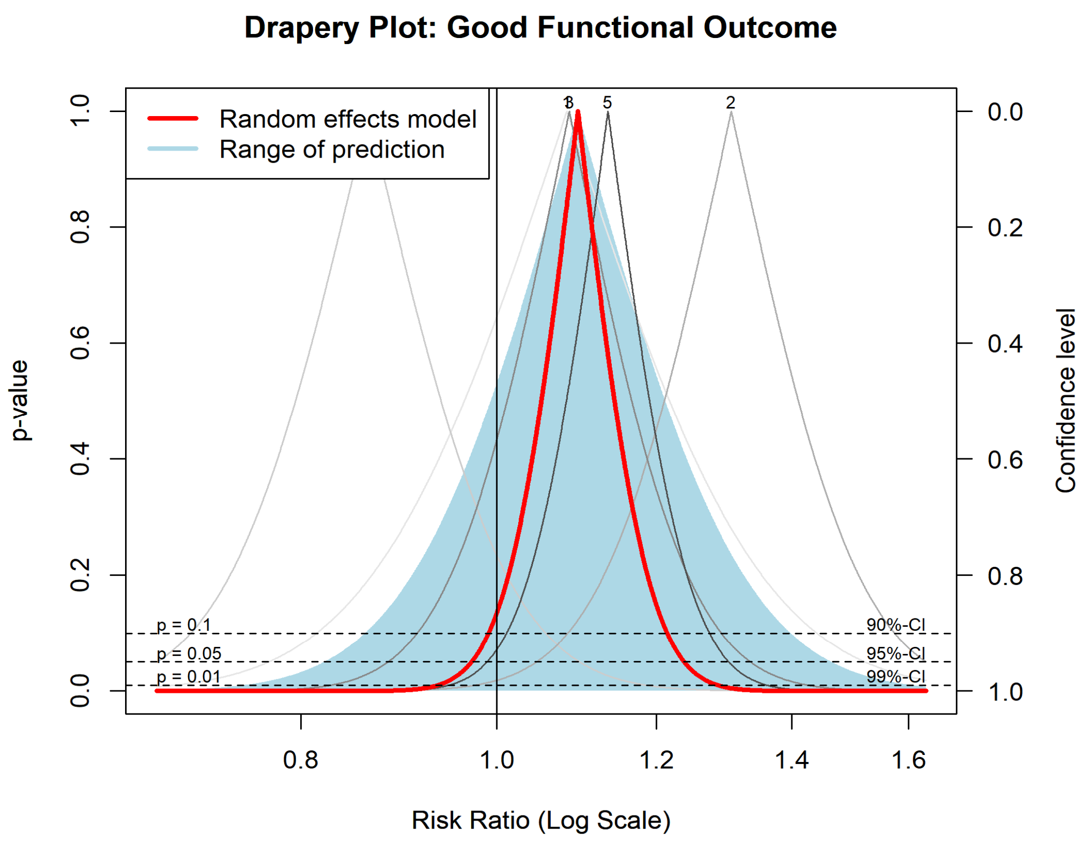

Legend: Drapery plot of good functional outcome.

Drapery plot illustrates the p-value functions, confidence levels, pooled random-effects estimate, and prediction range for a good functional outcome.

Supplementary Figure 10. Funnel plot

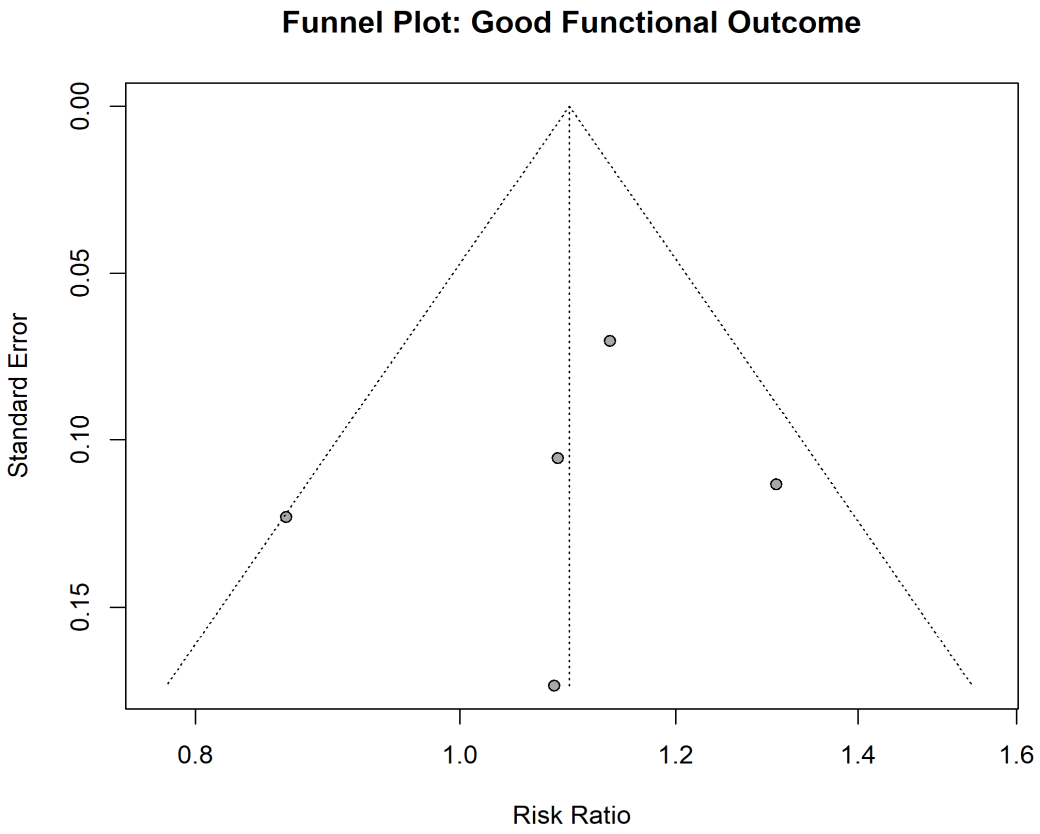

Legend: Funnel plot of good functional outcome.
Funnel plot assessing potential small-study effects and publication bias for the outcome of good functional outcome across the included studies.

Block B: Revascularization & Early Recovery (Supplementary Figs 11–20)

Supplementary Figs 11–15: Recanalization.

Supplementary Figure 11. Leave-One-Out Sensitivity Analysis

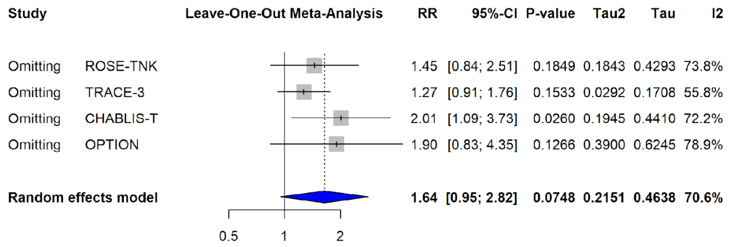

Legend: Leave-one-out sensitivity analysis of recanalization.
Leave-one-out sensitivity analysis showing the influence of sequential omission of individual studies on the pooled effect estimate for recanalization and evaluating the robustness of the overall findings.

Supplementary Figure 12. GOSH Analysis

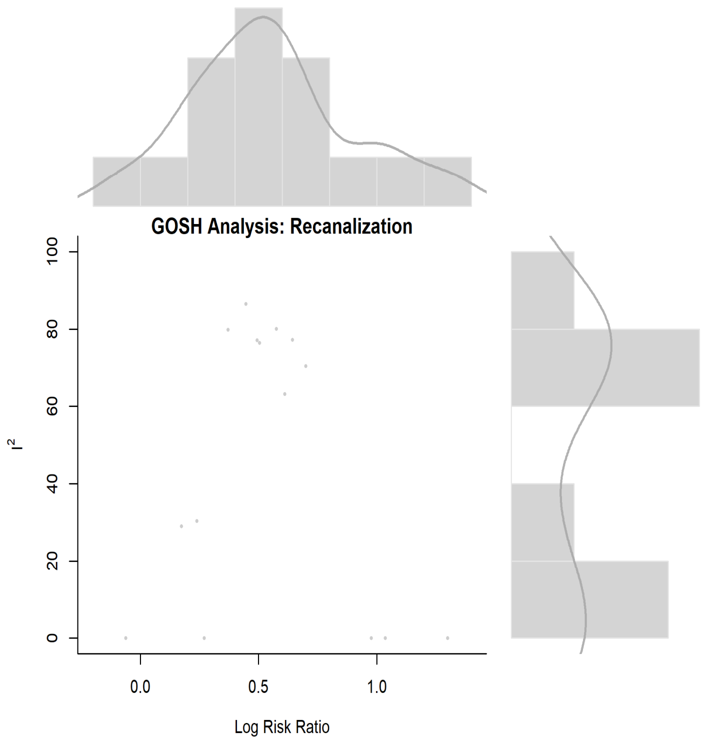

Legend: GOSH plot of recanalization.
Graphic display of study heterogeneity (GOSH) plot showing the distribution of pooled log risk ratios and heterogeneity estimates across all possible study subsets for recanalization.

Supplementary Figure 13. Baujat Plot

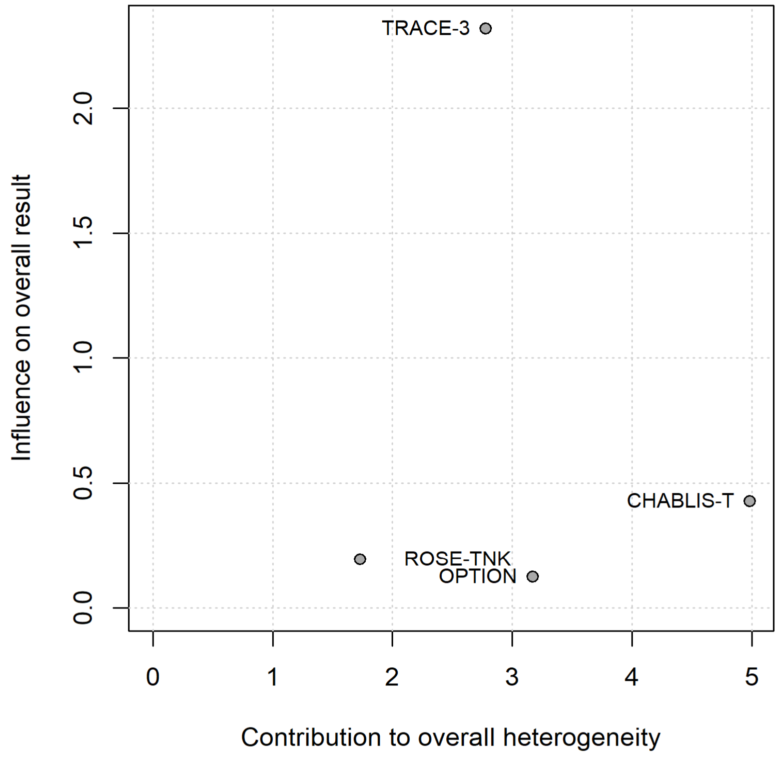

Legend: Baujat plot of recanalization.
Baujat plot identifying the relative contribution of each included study to overall heterogeneity and its influence on the pooled effect estimate for recanalization.**Supplementary**

Figure 14. p-value Functions (Drapery Plot)

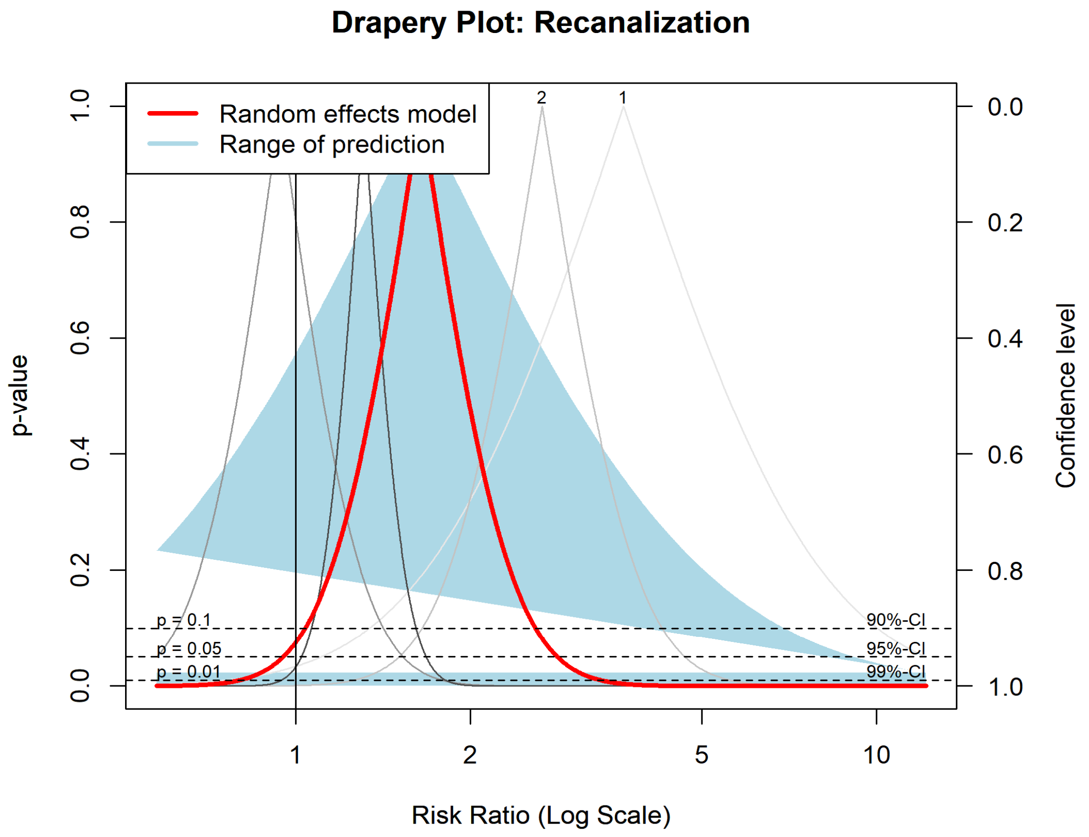

Legend: Drapery plot of recanalization.
Drapery plot illustrating the p-value functions, confidence levels, pooled random-effects estimate, and prediction range for recanalization.

Supplementary Figure 15. Funnel Plot

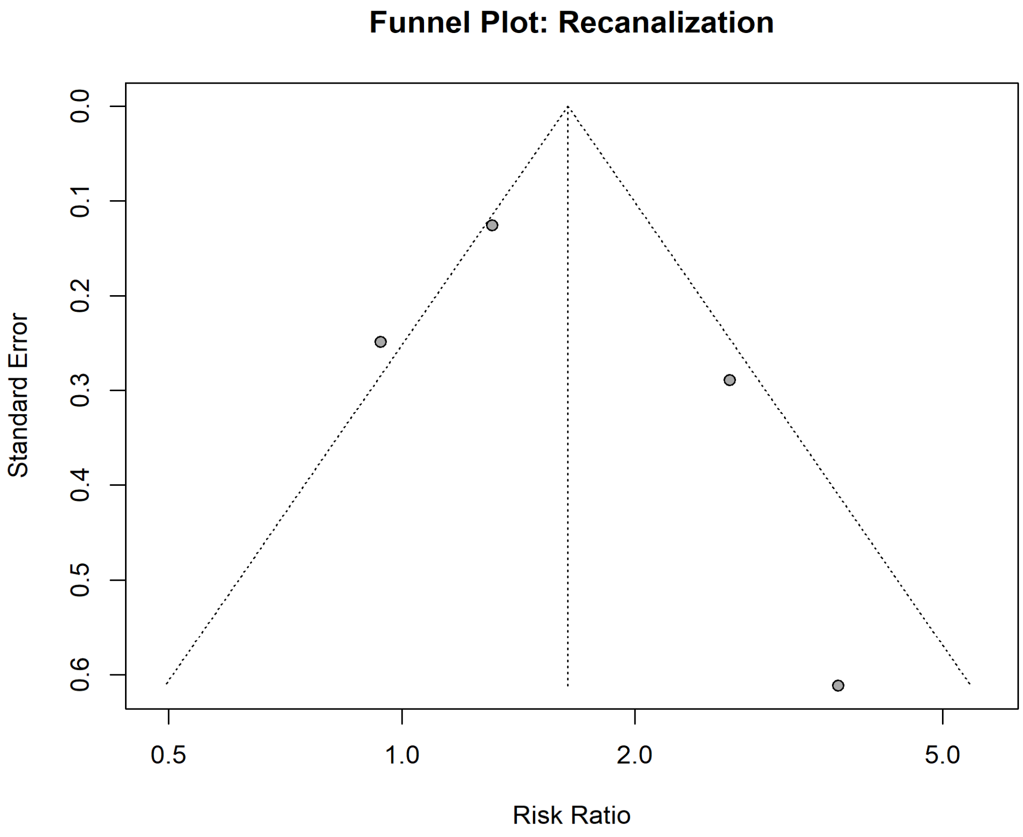

Legend: Funnel plot of recanalization.
Funnel plot assessing potential small-study effects and publication bias for the outcome of recanalization across the included studies.

Supplementary Figs 16–20: Early Neurological Improvement (ENI).

Supplementary Figure 16. Leave-One-Out Sensitivity Analysis

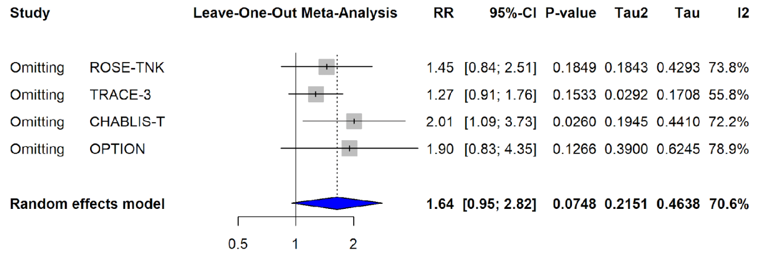

Legend: Leave-one-out sensitivity analysis showing the influence of sequential omission of individual studies on the pooled effect estimate for early neurological improvement and evaluating the robustness of the overall findings.

Supplementary Figure 17. GOSH Analysis

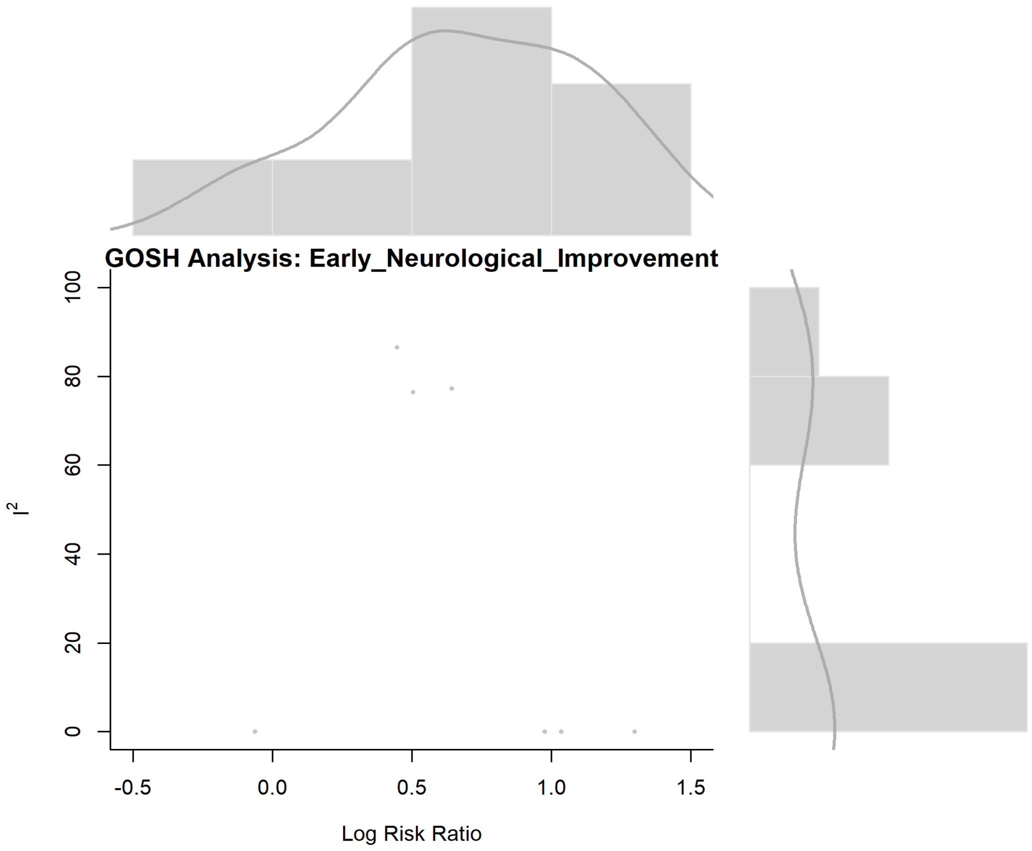

Legend: GOSH plot of early neurological improvement.
Graphic display of study heterogeneity (GOSH) plot showing the distribution of pooled log risk ratios and heterogeneity estimates across all possible study subsets for early neurological improvement.

Supplementary Figure 18. Baujat Plot

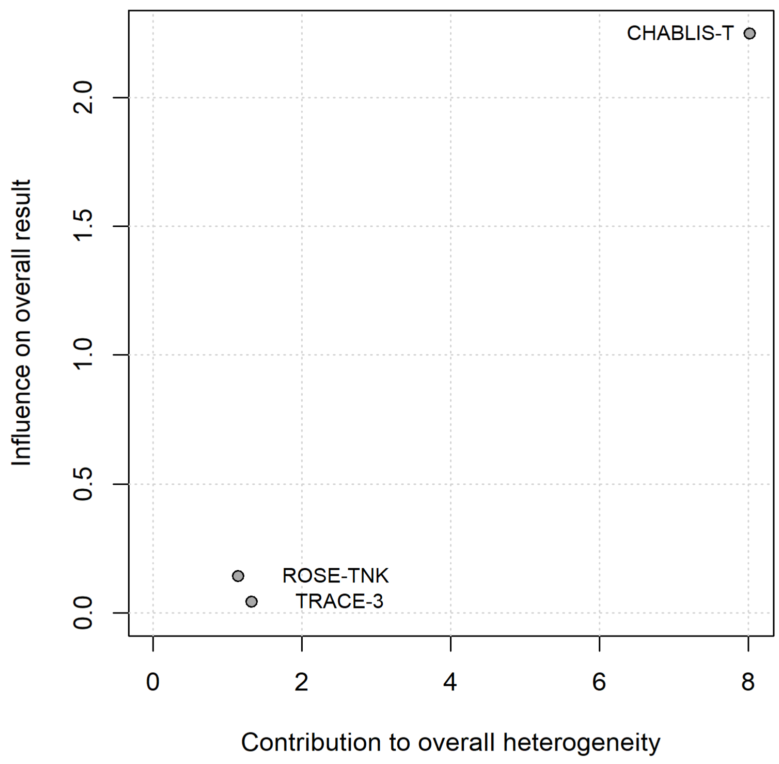

Legend: Baujat plot of early neurological improvement.
Baujat plot identifying the relative contribution of each included study to overall heterogeneity and its influence on the pooled effect estimate for early neurological improvement.

Supplementary Figure 19. p-value Functions (Drapery Plot)

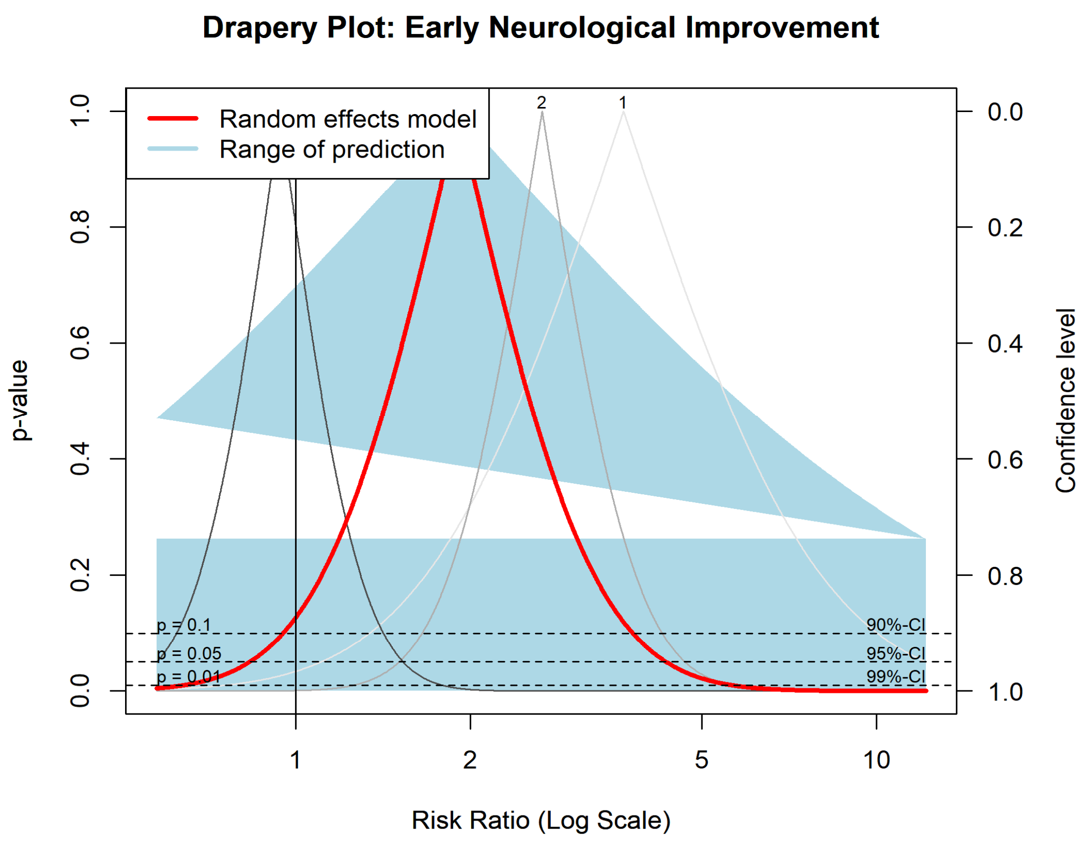

Legend: Drapery plot of early neurological improvement.
Drapery plot illustrating the p-value functions, confidence levels, pooled random-effects estimate, and prediction range for early neurological improvement.

Supplementary Figure 20. Funnel Plot

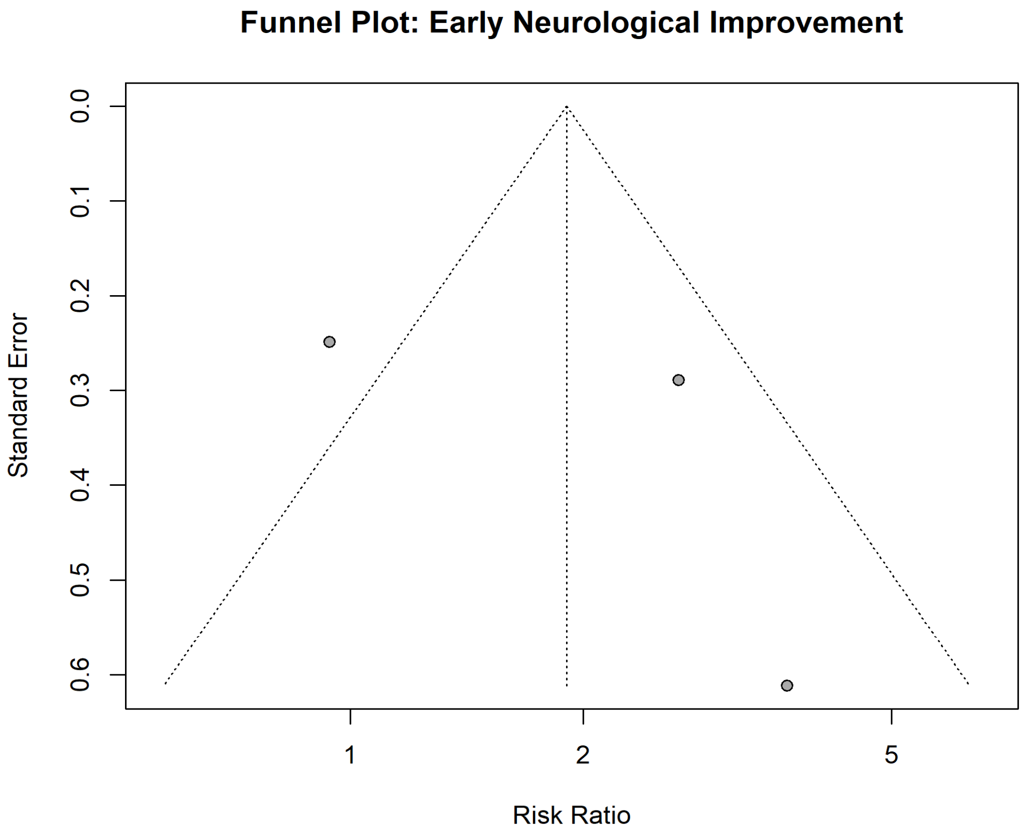

Legend: Funnel plot of early neurological improvement.
Funnel plot assessing potential small-study effects and publication bias for the outcome of early neurological improvement across the included studies.

Block C: Safety Outcomes (Figs 21–35)
Supplementary Figs 21–25: Symptomatic Intracranial Hemorrhage (sICH).

Supplementary Figure 21. Leave-One-Out Sensitivity Analysis

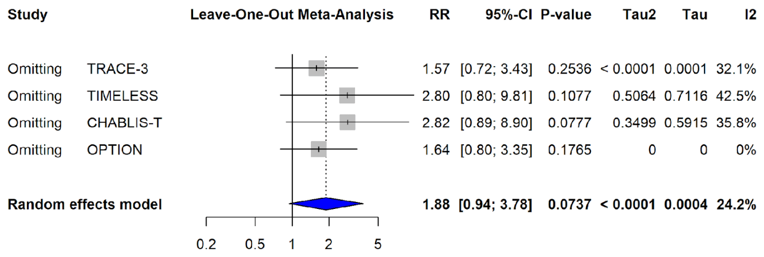

Legend: Leave-one-out sensitivity analysis of symptomatic intracerebral hemorrhage.
Leave-one-out sensitivity analysis showing the influence of sequential omission of individual studies on the pooled effect estimate for symptomatic intracerebral hemorrhage and evaluating the robustness of the overall findings.

Supplementary Figure 22. GOSH Analysis

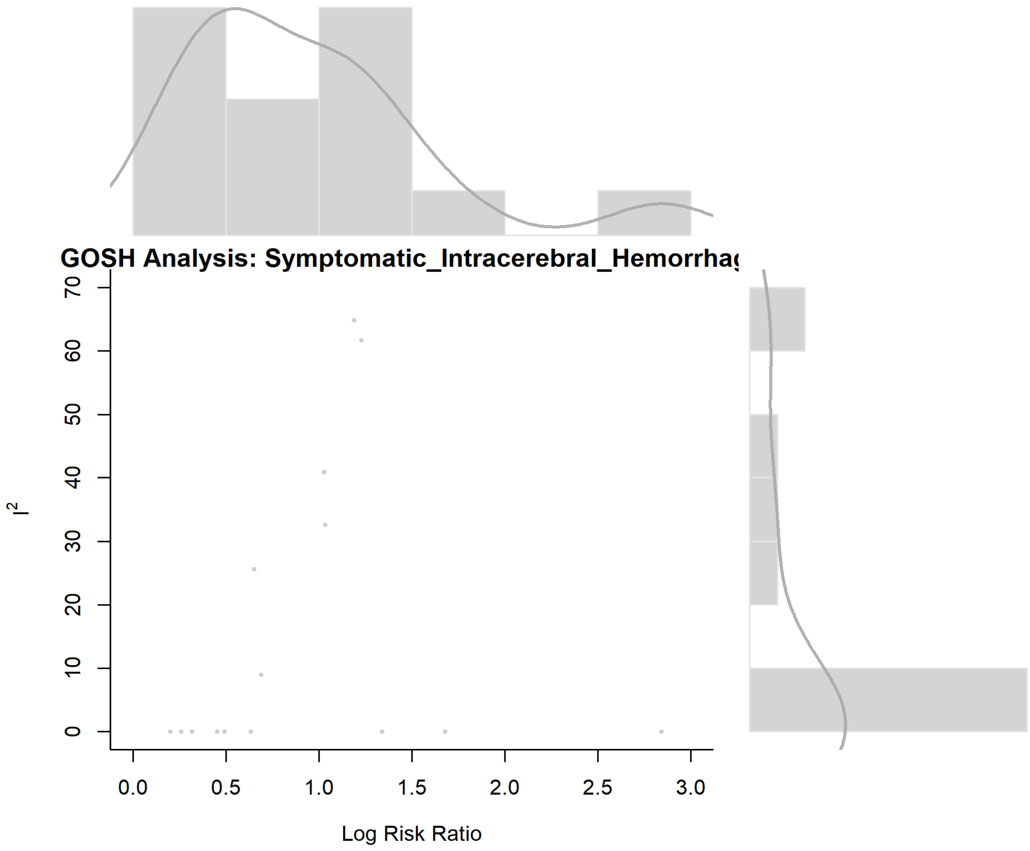

Legend: GOSH plot of symptomatic intracerebral hemorrhage.
Graphic display of study heterogeneity (GOSH) plot showing the distribution of pooled log risk ratios and heterogeneity estimates across all possible study subsets for symptomatic intracerebral hemorrhage.

Supplementary Figure 23. Baujat Plot

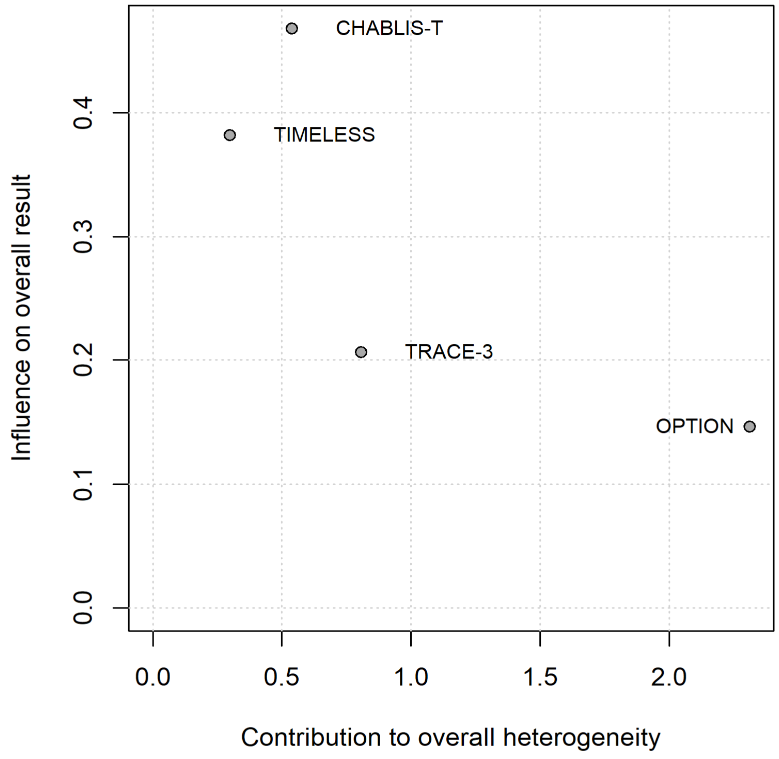

Legend: Baujat plot of symptomatic intracerebral hemorrhage.
Baujat plot identifying the relative contribution of each included study to overall heterogeneity and its influence on the pooled effect estimate for symptomatic intracerebral hemorrhage.

Supplementary Figure 24. p-value Functions (Drapery Plot)

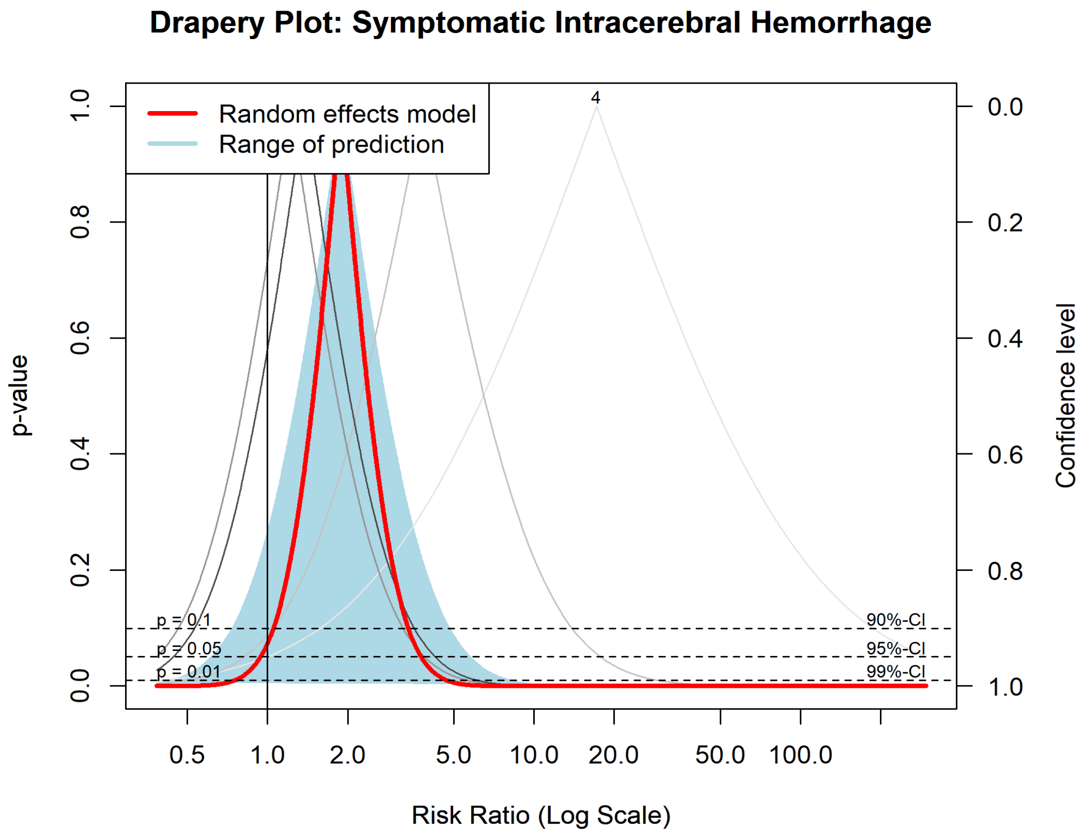

Legend: Drapery plot of symptomatic intracerebral hemorrhage.
Drapery plot illustrating the p-value functions, confidence levels, pooled random-effects estimate, and prediction range for symptomatic intracerebral hemorrhage.

Supplementary Figure 25. Funnel Plot

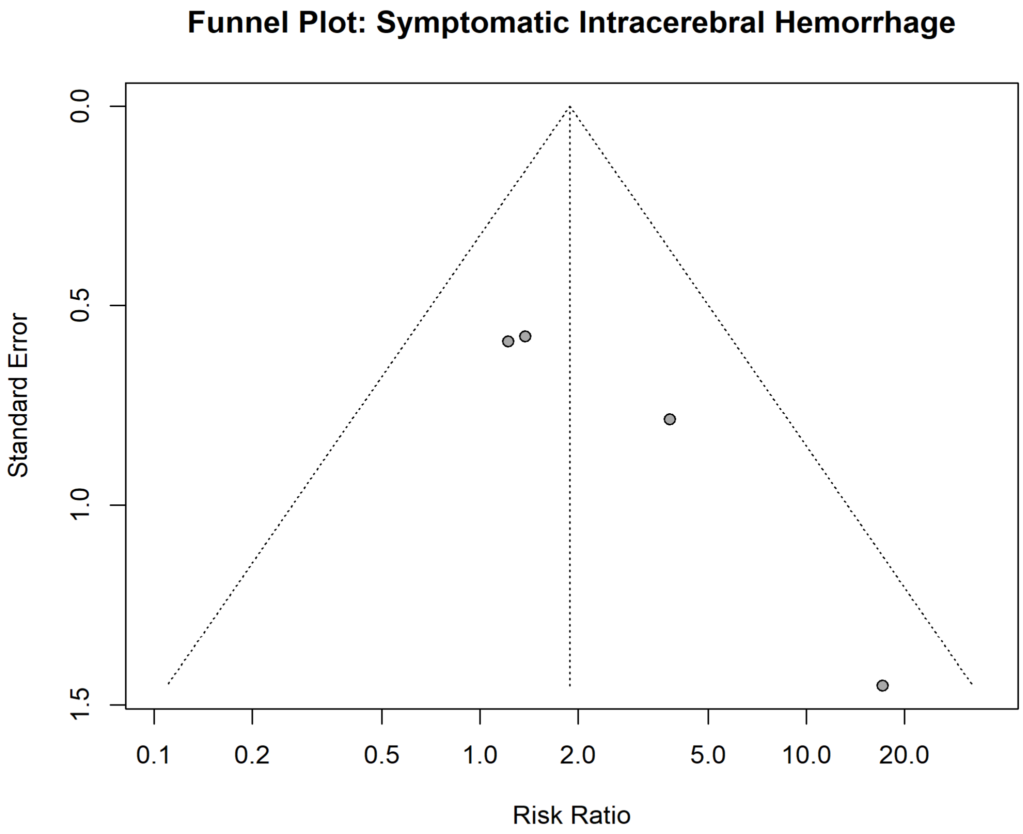

Legend: Funnel plot of symptomatic intracerebral hemorrhage.
Funnel plot assessing potential small-study effects and publication bias for the outcome of symptomatic intracerebral hemorrhage across the included studies.

Supplementary Figs 26–30: Moderate-to-Severe Systemic Bleeding.
Supplementary Figure 26. Leave-One-Out Sensitivity Analysis

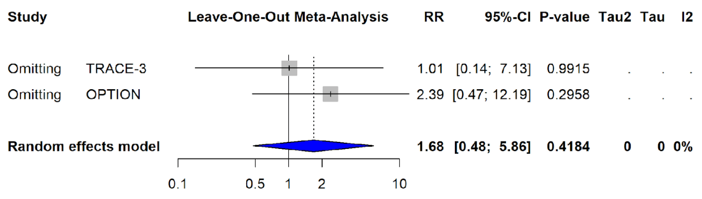

Legend: Leave-one-out sensitivity analysis of moderate or severe systemic bleeding.
Leave-one-out sensitivity analysis showing the influence of sequential omission of individual studies on the pooled effect estimate for moderate or severe systemic bleeding and evaluating the robustness of the overall findings.

Supplementary Figure 27. GOSH Analysis

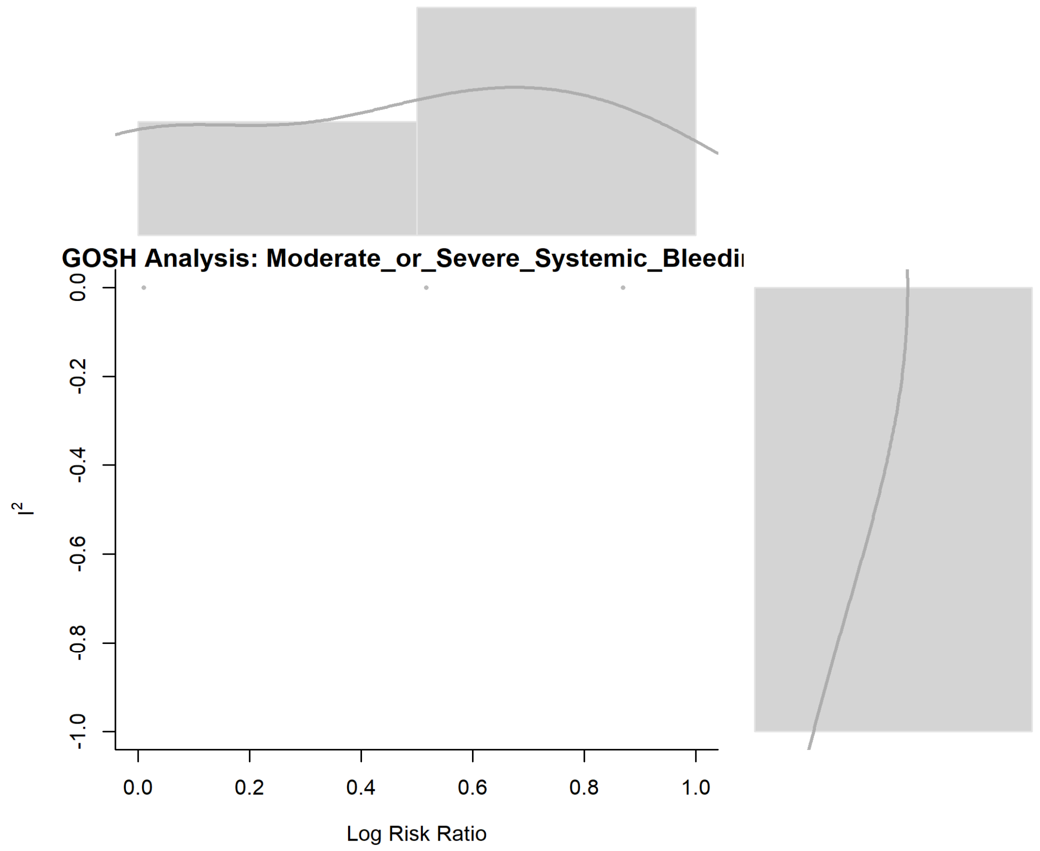

Legend: GOSH plot of moderate or severe systemic bleeding.
Graphic display of study heterogeneity (GOSH) plot showing the distribution of pooled log risk ratios and heterogeneity estimates across all possible study subsets for moderate or severe systemic bleeding.

Supplementary Figure 28. Baujat Plot

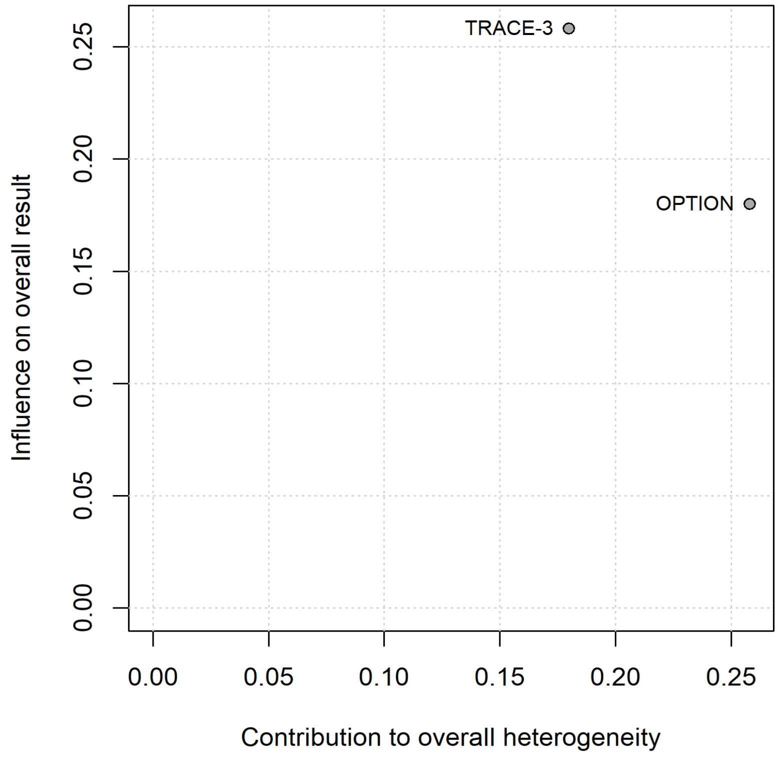

Legend: Baujat plot of moderate or severe systemic bleeding.
Baujat plot identifying the relative contribution of each included study to overall heterogeneity and its influence on the pooled effect estimate for moderate or severe systemic bleeding.

Supplementary Figure 29. p-value Functions (Drapery Plot)

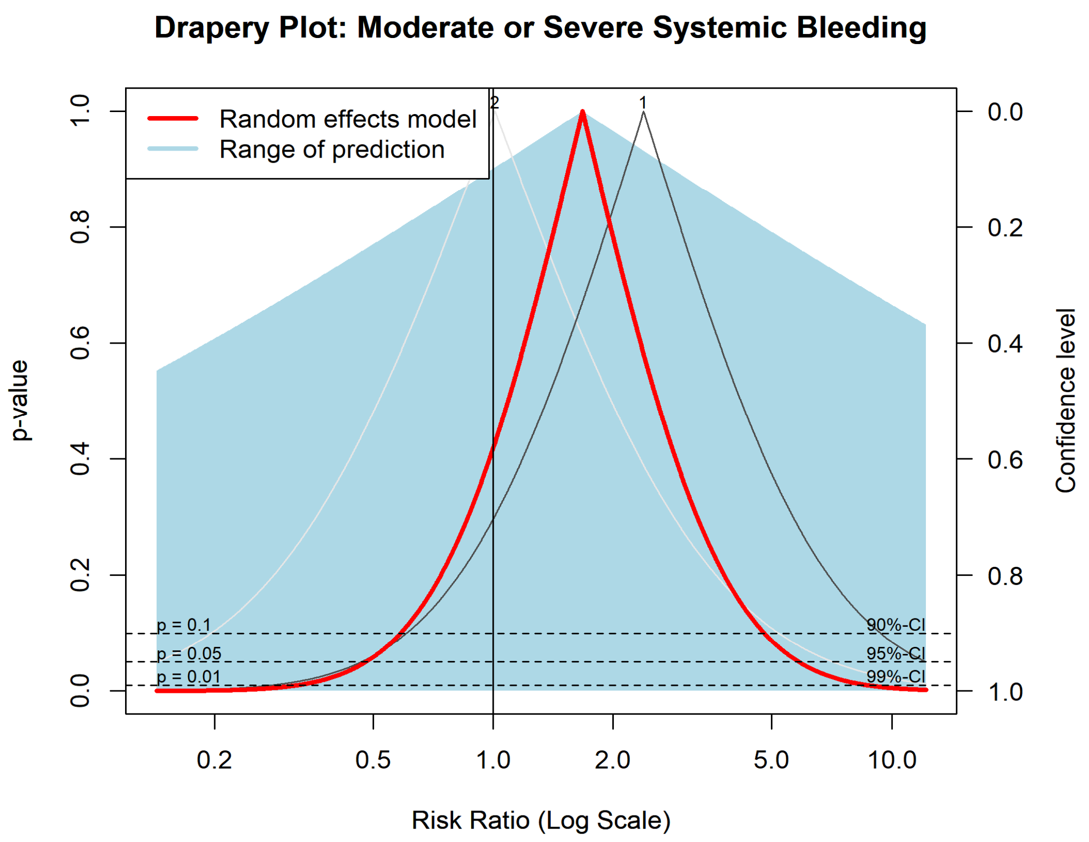

Legend: Drapery plot of moderate or severe systemic bleeding.
Drapery plot illustrating the p-value functions, confidence levels, pooled random-effects estimate, and prediction range for moderate or severe systemic bleeding.

Supplementary Figure 30. Funnel Plot

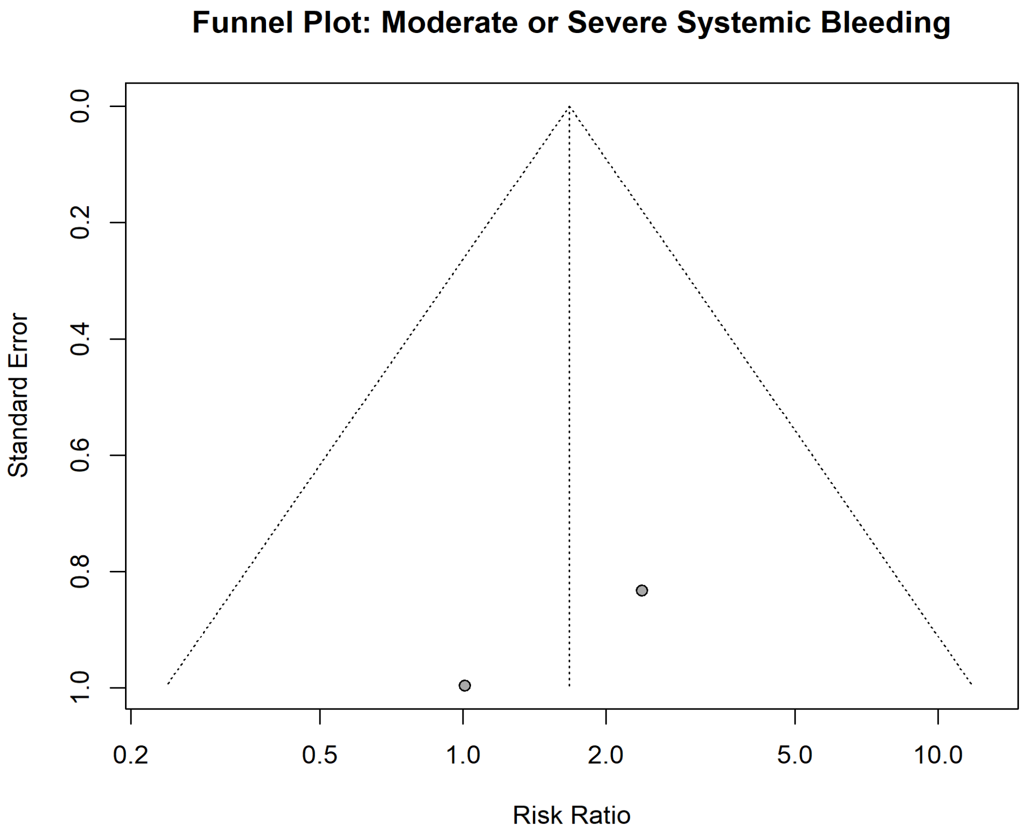

Legend: Funnel plot of moderate or severe systemic bleeding.
Funnel plot assessing potential small-study effects and publication bias for the outcome of moderate or severe systemic bleeding across the included studies.

Supplementary Figs 31–35: **90-Day All-Cause Mortality.**

Supplementary Figure 31. Leave-One-Out Sensitivity Analysis

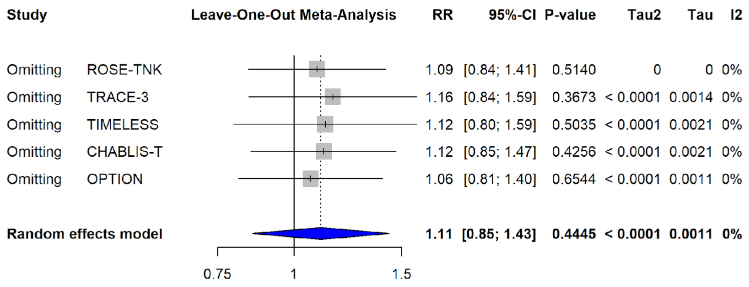

Legend: Leave-one-out sensitivity analysis of death within 90 days.

Leave-one-out sensitivity analysis showing the influence of sequential omission of individual studies on the pooled effect estimate for death within 90 days and demonstrating the stability of the overall findings.

Supplementary Figure 32. GOSH Analysis

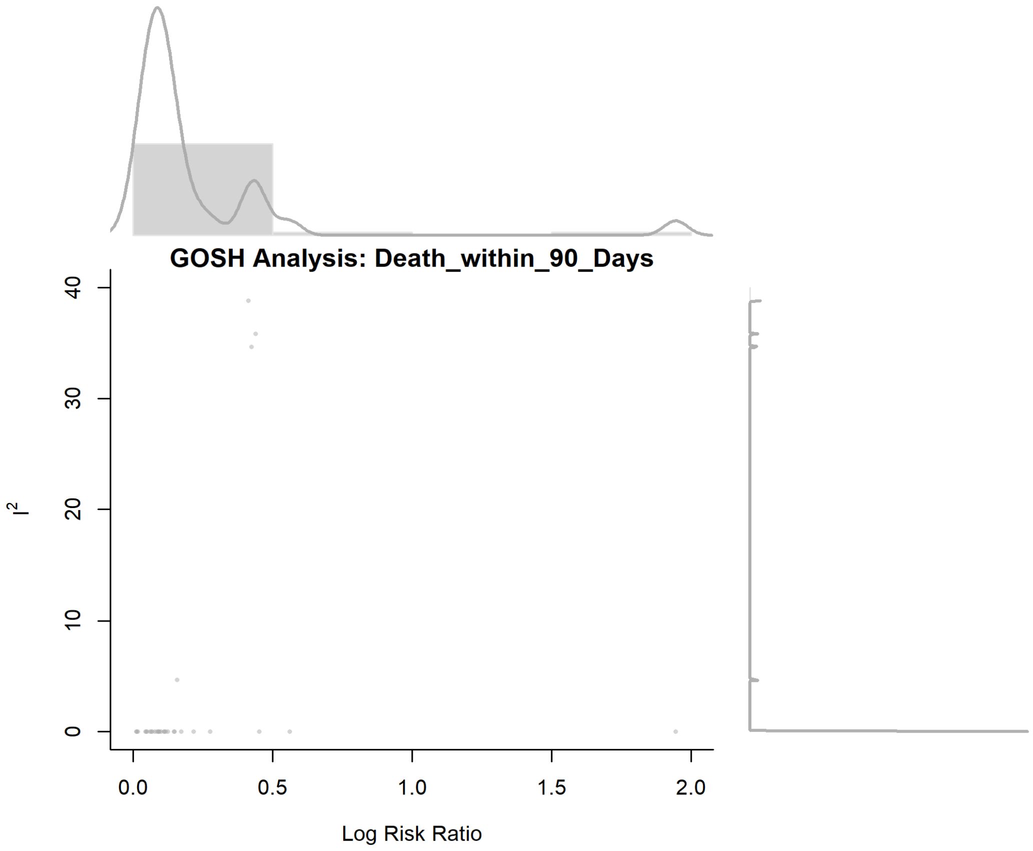

Legend: GOSH plot of death within 90 days.
Graphic display of study heterogeneity (GOSH) plot showing the distribution of pooled log risk ratios and heterogeneity estimates across all possible study subsets for death within 90 days.

Supplementary Figure 33. Baujat Plot

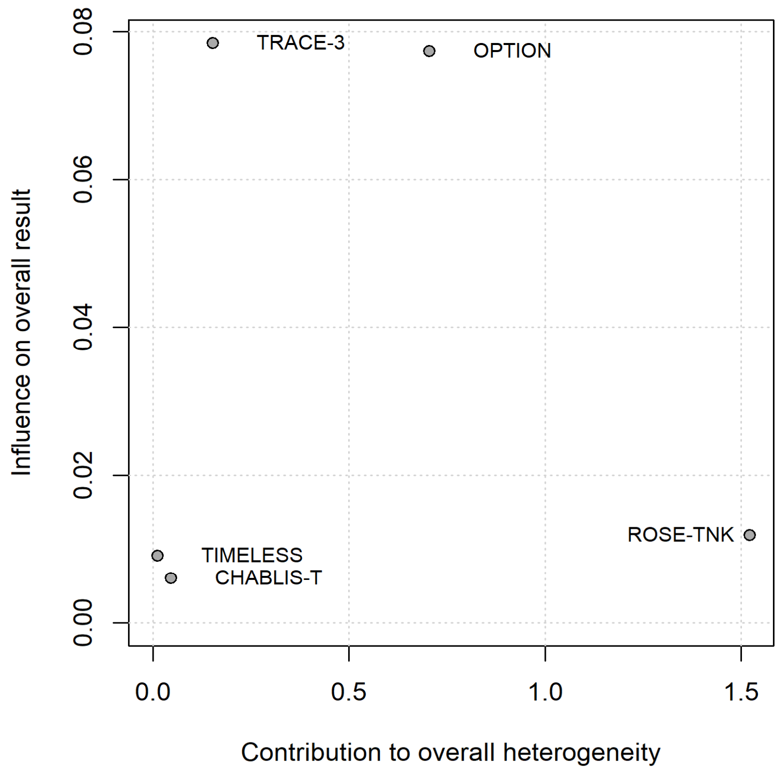

Legend: Baujat plot of death within 90 days.
Baujat plot identifying the relative contribution of each included study to overall heterogeneity and its influence on the pooled effect estimate for death within 90 days.

Supplementary Figure 34. p-value Functions (Drapery Plot)

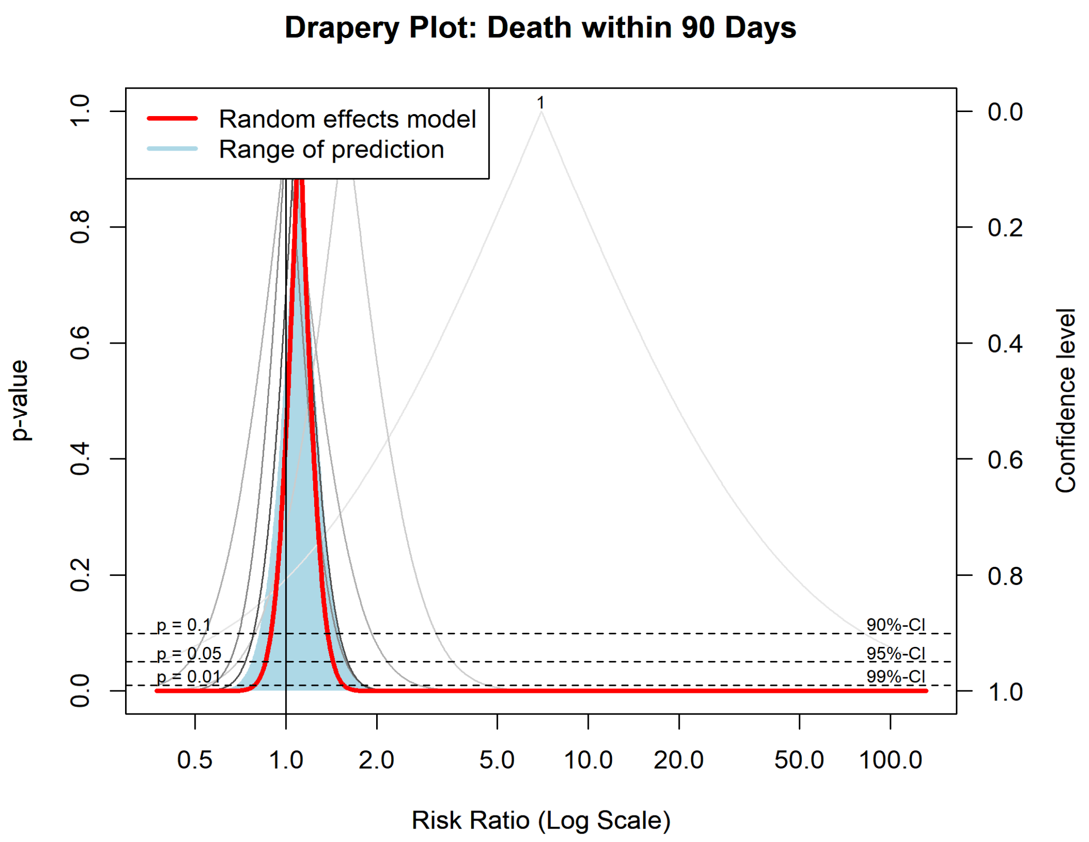

Legend: Drapery plot of death within 90 days.
Drapery plot illustrating the p-value functions, confidence levels, pooled random-effects estimate, and prediction range for death within 90 days

Supplementary Figure 35. Funnel Plot

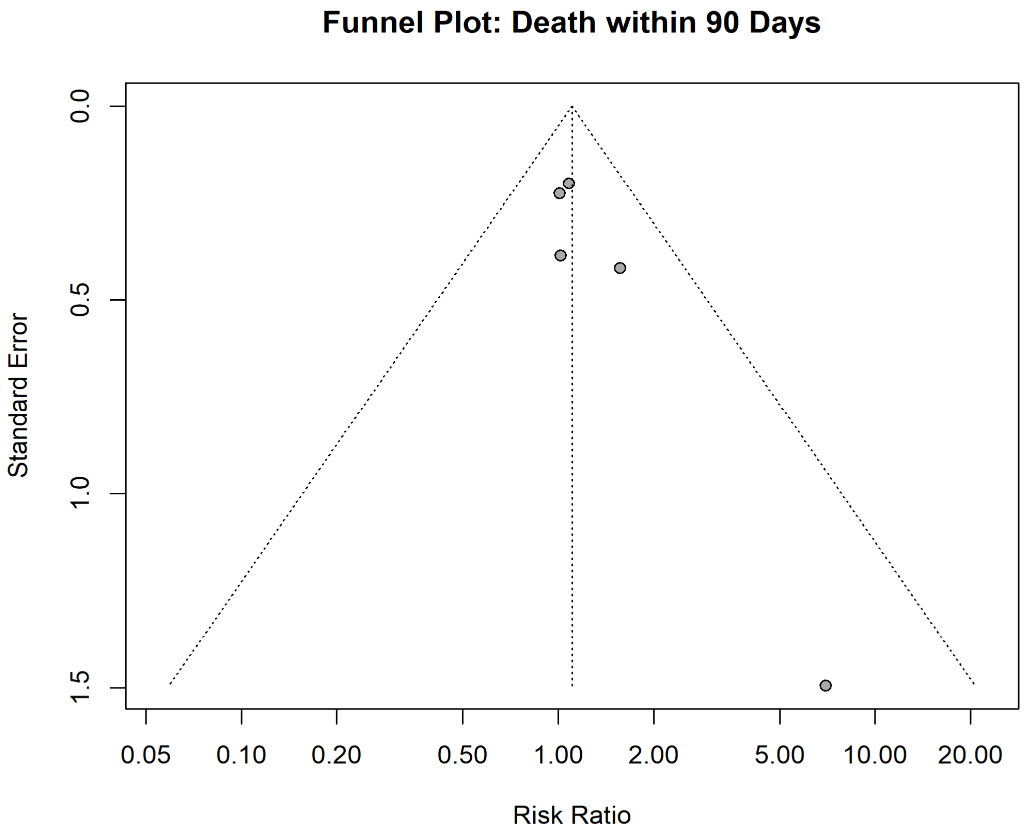

Legend: Funnel plot of death within 90 days.
Funnel plot assessing potential small-study effects and publication bias for the outcome of death within 90 days across the included studies.
